# “How can you kiss and touch this child and show affection towards her? What kind of woman are you?”: Provider perspectives on stigma towards native and ethnic minority street-connected youth in the Republic of Georgia

**DOI:** 10.1371/journal.pone.0286710

**Published:** 2023-06-02

**Authors:** Shorena Sadzaglishvili, Teona Gotsiridze, Ketevan Lekishvili, Rey Flores, Jane Hereth, Alida Bouris

**Affiliations:** 1 Ilia State University, Tbilisi, Georgia; 2 Chicago Center for HIV Elimination, University of Chicago Medicine, Chicago, Illinois, United States of America; 3 University of Wisconsin Helen Bader School of Social Welfare, Milwaukee, Wisconsin, United States of America; 4 Crown Family School of Social Work, Policy and Practice, University of Chicago, Chicago, Illinois, United States of America; Tallinn University: Tallinna Ulikool, ESTONIA

## Abstract

The Republic of Georgia has experienced a rapid growth in the number of youth working and/or living on the street (YWLS). Although research indicates that YWLS are highly stigmatized, few studies have examined perceptions of stigma among Georgian social service providers who serve YWLS. We conducted in-person in-depth interviews with key informants recruited from governmental institutions and social service organizations in Tbilisi and Rustavi, two large urban areas. A semi-structured interview guide was used to explore provider perspectives on the social contexts surrounding the delivery of services to YWLS. Trained coders conducted a thematic analysis of the data in Dedoose. Twenty-two providers (68% female; 32% male) were interviewed, representing diverse professional roles. Providers perceived that YWLS are subjected to strong public stigma and social exclusion at multiple social-ecological levels, with Roma and Kurdish-Azeri youth experiencing the strongest levels of social hostility, discrimination, and exclusion. Providers perceive that these dynamics prevent YWLS from developing trusting relationships with social service, health and educational institutions. Furthermore, we find that providers report encounters with courtesy stigma, i.e., stigma directed towards the people who serve or are associated with a stigmatized group, when working with YWLS, especially those from ethnic minority groups, which they characterize as a stressor. At the same time, we find that some providers reported negative stereotypes about ethnic minority YWLS. While campaigns have targeted public awareness on the plight of YWLS, study findings suggest that additional efforts are needed to address stigma directed towards YWLS, with a specific need to address stigma directed towards ethnic minority young people who work and/or live on the street.

## Introduction

Young people aged 10 to 19 years old who spend most of their time working and/or living on the street experience high rates of stigma and discrimination, as well as social and economic exclusion [1–4]. Among youth working and/or living on the street (YWLS), stigma and discrimination are key correlates of poor health and mental health outcomes [2]. Numerous studies in low-, middle-, and high-income countries have found that YWLS are vulnerable to negative health and social outcomes, including violence and victimization [5], economic and sexual exploitation [6], poor mental health [7], substance abuse [8], and sexually transmitted infections, including HIV/AIDS [9]. In the Republic of Georgia, YWLS are a relatively new phenomenon [10], with studies estimating that more than 1,600 youth work and/or live on the streets [11]. Although some research has examined the social, health, and economic needs of YWLS in Georgia [4], few studies have explored how social service providers perceive, manage, and respond to the stigma directed towards youth who live and/or work on the street. The present study helps to address this gap by examining how social service providers who serve YWLS in Tbilisi and Rustavi, two major cities home to an estimated 1,094 YWLS [11], perceive the social contexts in which they deliver services to YWLS.

Worldwide, more than 100 million young people work and/or live on the street [12]. The majority are concentrated in urban centers and engage in the street economy seasonally or all year [4]. Prior research has documented how Georgia’s transitional macro-social processes led to increased poverty, unemployment, and homelessness for numerous families, driving many children to the streets [11]. In addition, internal armed conflicts and the war with Russia forced the migration of families from Georgia’s conflict zones in Abkhazia and South Ossetia to urban centers; once displaced, many families were forced to live in deteriorated conditions, which increased family stress and resulted in a number of children living and working on the streets [11]. In Georgia, YWLS represent multiple ethnic and linguistic groups, including ethnic Georgians, Azerbaijani Kurds, two Romani groups that speak distinct languages, refugees and internally displaced persons from Armenia, South Ossetia and Abkhazia, and youth from European and neighboring countries, e.g., Greece, Ukraine [4, 10]. Since 2007, there has been a notable increase in the numbers of YWLS and families from Azerbaijan, identified as Azerbaijani or Azeri Kurds, who lack documentation and the ability to access many health and social services [4].

The growing number of YWLS in Georgia is a significant social welfare issue, with public demands and governmental efforts to decrease the population [4, 10]. Current social service delivery for YWLS in Georgia focuses on preventing youth from entering street life and on reintegrating young people into mainstream social life [4]. To support these goals, the Georgian Ministry of Labor, Health, and Social Affairs has established mobile groups to serve YWLS. These groups are based in major urban centers and led by State Senior Social Workers, with allied health professionals like psychologists, peer educators, logistics officers, and the managers of social service organizations. Together, these teams conduct street-based outreach and formally assess and enroll youth in appropriate service programs, such as day care services and 24-hour shelters [13]. In addition, non-governmental organizations (NGOs) and international groups, such as World Vision, UNICEF and Caritas, conduct needs assessments, deliver services, and advocate for policies for YWLS and their families [4]. Despite the prioritized governmental protection of YWLS, they remain a highly stigmatized population [4, 14].

Much of the scholarly work on stigma draws from Erving Goffman’s [15] seminal work on stigma, where he elucidated how stigma operates as a discrediting mark of the body, character, and/or status. In describing the process of stigma, Goffman [15] described how stigmatized persons are viewed as “not quite human” (p. 15), which is used to justify various forms of social exclusion, discrimination, and marginalization [16–18]. Link and Phelan [16] extend Goffman’s work on stigma by explicating the interrelated processes of stigma, starting with the distinguishing and labeling of human differences and the attachment of negative stereotypes to identified differences. Together labeling and stereotyping processes serve to separate the non-stigmatized from the stigmatized, creating a clear “us” and “them” [16]. This separation leads to status loss and discrimination among the stigmatized, highlighting the role of social power in the stigma process [16]. Although a single attribute can be the sole reason to experience stigma, people may hold multiple devalued attributes [15, 16]. Recent scholarship has focused on intersectional stigma, i.e., the presence of multiple devalued social markers, such as race, class, and sexual identity, that interact to produce layered experiences of stigma and discrimination [19–22].

Public stigma towards socially stigmatized groups is well-documented [23, 24]. Across studies, public stigma has been found to have a negative impact on the well-being of people living with mental illness [25, 26], sexual and gender minority persons [27], people who use drugs [28], people living with HIV [29], people engaged in sex work and the street economy [30], and people who are immigrants and refugees [31]. Because stigma is socially produced, public stigma can be transferred to people affiliated with stigmatized groups, a process referred to as courtesy stigma, including health and social service providers [32–34]. In a qualitative study with providers serving sex workers, providers described encountering courtesy stigma from friends and family, other providers, and the broader public [32]. To manage courtesy stigma, providers used impression management strategies, such as distinguishing how they were different from sex workers and intentionally framing sex workers as victims who deserve sympathy [32]. However, these strategies were not available to providers with shared histories of sex work, poverty, and marginalization [32]. Furthermore, courtesy stigma negatively impacted providers’ well-being and contributed to an overall stressful work environment [32].

Beyond these dynamics, providers exposed to stigmatizing messages about the groups they serve may come to adopt negative beliefs through the course of their work [35–39]. These beliefs can persist even among providers with specialized education and training [40, 41], highlighting the need to address stigma at multiple educational and professional levels. Furthermore, provider stigma can negatively impact willingness to engage in services [37, 42], the quality of provider care [38, 39], and the physical and mental health of stigmatized groups [35]. When institutionalized within an organization, provider stigma can operate as a form of structural stigma [43]. According to Livingston and Boyd [44], these forms of stigma, which operate at the macro level, encapsulate the “rules, policies, and procedures of private and public entities in positions of power that restrict the rights and opportunities” of a stigmatized group.

To date, little research has examined how Georgian providers perceive and respond to stigma directed at the youth they serve, and how such experiences shape their own perceptions and work. One exception is a recent report from UNICEF, where interviews with social service providers, YWLS and their families in Georgia and Azerbaijan revealed high levels of public stigma towards YWLS [4]. Another NGO report presenting results from a survey with 300 YWLS discussed high rates of public stigma towards YWLS, but did not interview social service providers [11]. To date, most research examining social service providers’ perspectives on stigma in the Georgian context has focused on women who use drugs. For example, in a study with 34 health service providers (i.e., physicians, nurses, psychologists, and drug counselors) serving Georgian women who use drugs, the authors concluded that Georgian women were “twice stigmatized”: first by Georgian society and then by health service providers, who viewed them “as failed mothers, wives or daughters” [28].

In addition, several studies have noted high rates of interpersonal and systemic discrimination [45–48] directed at ethnic minorities and immigrants in Georgia, including YWLS [4, 11]. Romani communities, in particular, experience economic hardship due to discrete and indiscrete forms of employment discrimination in hiring and wage allocation [49], leading them to engage in street economies to support themselves and their families [50]. Stigma, discrimination, and hostility towards Romani peoples have been documented in numerous European and non-European countries [49, 51–56], including Georgia [50] and other former Soviet Republics [57, 58]. Antiziganism, as defined by the European Commission Against Racism and Intolerance, is defined as a “specific form of racism” that is founded on an ideology of “racial superiority, a form of dehumanization and institutional racism nurtured by historical discrimination, which is expressed, among others, by violence, hate speech, exploitation, stigmatization and the most blatant kind of discrimination.” Romaphobia, in turn, has been defined as “an invisible, structural form of racism built on the denial of racism against Romanis,” which results in systematic discrimination and a type of “structural blindness” towards the social, economic, and political barriers encountered by Romani peoples [51]. Scholars also have discussed how structural stigma and violence occur to non-Georgians due to state-building from previous conflicts between cultural groups [47]. For example, the Ossetian conflict enabled militaries to engage in “ethnic cleansing,” which created deep-seated animus between Georgians and non-Georgians that has overflowed into public attitudes and beliefs [59]. As such, it is possible that providers may consciously or unconsciously endorse these attitudes in their work with YWLS from ethnic minority groups. Indeed, UNICEF’s report discussed provider stigma towards ethnic-minority YWLS, leading to a call for multi-lingual providers, minority integration policies, and stigma-reduction programs [4].

Finally, providers may themselves hold visible or hidden traits that are stigmatized, which may subject them to similar stigmatizing processes as their beneficiaries. This may be especially relevant in work with YWLS, where peer workers draw on their lived experiences to conduct outreach, distribute resources, and increase access to street-involved youth. A growing body of research is examining how the lived experiences of human service providers shape experiences and responses to stigma, as well as provider’s roles, attitudes and responsibilities towards service users [60–63]. These studies indicate that while providers draw on their own lived experience to improve service engagement and delivery with affected populations [61], some also endorse stigmatizing beliefs and attitudes, thereby creating within group stigma [62].

## Methods

This qualitative exploratory study was conducted during the formative phase of See the Future in Us, a multi-method study examining the social networks and HIV-prevention needs of YWLS [10]. During this exploratory phase, we conducted individual in-depth interviews with key informants employed in social service organizations working with YWLS in Tbilisi and Rustavi. The purpose of the interviews was to understand provider perspectives on the social networks and HIV prevention needs of YWLS, their experiences delivering services to YWLS, and their recommendations for developing trusting relationships with YWLS. Key informants included social workers, psychologists, logistics officers who supported mobile outreach teams, managers of social service organizations, and peer educators who were young adults with a prior history of living and/or working on the street.

### Recruitment

We developed a sampling frame based on the 10 known governmental and non-governmental organizations that provide services to YWLS in Tbilisi and Rustavi. From this list, we used a purposive sampling method to identify and recruit potentially eligible key informants (KIs) who worked with YWLS in each agency. KIs were eligible to participate if they (1) were 18 years or older, (2) worked in an agency providing services to YWLS; and (3) had direct contact with YWLS through their work. All eligible staff in each agency were contacted via telephone and invited to participate in an individual interview. During recruitment, the local research team explained the purpose of the study and all participants provided written informed consent. In total, 95% of all identified and eligible staff were recruited into the study. None of the study participants had an official relationship with the study and were not involved in developing the study or interpreting its results.

In total, 22 key informants were interviewed. Of these, 68% identified as female and 32% identified as male. The majority (86.4%) identified as ethnic Georgian, with smaller numbers identifying as Azeri-Kurdish (9.1%) and Roma (4.5%). On average, providers were 32.73 years old (SD = 8.07: Range 20–50 years). The majority of respondents identified as social workers (n = 6), followed by psychologists (n = 5) and peer educators (n = 5), managers [who also act as supervisors] (n = 4), and logistics offers, who act as drivers/mobile health officers (n = 2).

### Data collection

After providing consent, KIs participated in a semi-structured in-depth interview that used open-ended questions to explore provider perspectives in three key domains: (1) the social network characteristics of YWLS, (2) youth’s involvement in substance use and sexual behaviors that may heighten the likelihood for HIV transmission, and (3) the social contexts of youth engagement and service delivery, e.g., where youth congregate in the city, youth’s connections to teachers, social service providers, social workers, and other institutional officials, their experiences delivering services to YWLS, and how to develop trusting relationships with YWLS. The guide was informed by the extant literature on YWLS in Georgia [11] and by a community advisory board comprised of key stakeholders who serve YWLS. Interviews were conducted by trained Master’s- and doctoral-level social workers in Georgian. On average, interviews lasted for approximately 100 minutes (Range: 90 to 120 minutes). Each interview was audio recorded and then a written transcript was produced. In addition, interviewers completed analytic memos after each interview, capturing key qualitative impressions in the moment [64]. All transcripts were de-identified, and audio recordings were securely destroyed after the transcripts were checked for accuracy. No research incentives were provided. All study protocols were approved by the Ilia State University Ethics Committee on June 26, 2018 (R/333-18).

### Data analysis

Written transcripts were uploaded into Dedoose, an online, computer-assisted qualitative data analysis software program [65]. The team used a systematic and iterative approach to code the data following methods described by Saldaña [64]. First, the Georgian team (SS, TG, and KL) developed a preliminary codebook based on the initial interview guide [64]. The codebook was then refined based on analytic memos completed after each interview, which identified emergent themes and categories related to stigma across the 22 interviews [64]. Authors SS, TG, KL, and AB then discussed stigma concepts based on foundational [15] and contemporary scholarship on stigma [16, 24, 43]. To strengthen rigor, each transcript was then coded by three independent coders [66] (SS, TG, and KL). To help establish interrater reliability, the coders analyzed a single transcript in Dedoose [65]. The coders then met to discuss the first round of coding, revise the codebook, and to discuss areas of disagreement in the coding. After establishing a final codebook, the team then proceeded to analyze the transcripts using descriptive coding, applying codes to select passages in each transcript. SS, TG, and KL translated each excerpt to English following methods outlined in the Euro-Reves project, a forward-backward translation method that focuses on ensuring linguistic, cultural and conceptual equivalence [67]. For each coded passage, the coders included the professional role of the respondent and the respondent number. Authors SS, TG, KL and AB then created an analytic matrix mapping the selected codes on to identified dimensions of stigma. The team then discussed the codes and themes with the other authors, with the whole team elucidating themes and subthemes related to dimensions and manifestations of stigma.

### Positionality

Three members of the research team are based in Georgia (SS, KL, TG) and others in the United States (RF, JH, AB). While no members of the team have personal histories of living or working on the street, all are social service providers who share study participants’ experiences of working with young people and families experiencing homelessness or displacement, as well as other populations who experience stigma and discrimination (e.g., immigrants, sexual and gender minorities, people who inject drugs, and people living with HIV). Throughout the data collection and analysis process, we reflected upon our positionality as insiders and/or outsiders and engaged in dialogue to address possible sources of misunderstanding, misinterpretation, and bias when analyzing and interpreting study findings. This process was especially useful when discussing how providers described manifestations of antiziganism, ethnic discrimination and cultural racism [50] towards ethnic minority YWLS in Georgia, as this manifestation of stigma is both similar and distinct from racism within the United States.

## Results

We identified five themes and five subthemes. The primary themes are: (1) Already labeled objects who spoil everything: Public and institutional stigma directed towards YWLS; (2) Dirty and despicable criminals: Intersectional stigma for ethnic minority YWLS; (3) Provider stereotypes about ethnic minority youth: Labeling, stereotyping and separating “us” from “them;” (4) Youth fear, anxiety, and distrust: Provider perspectives on how public stigma affects YWLS; and (5) Difficult working environments: Provider encounters with courtesy stigma. Each theme and any relevant subthemes are discussed below, with illustrative quotes from interviews along with the professional role of each respondent.

### Theme 1. Already labeled objects who spoil everything: Public and institutional stigma directed towards YWLS

Across interviews, providers described a social environment characterized by strong public stigma towards YWLS. One provider described societal attitudes towards YWLS: *“They are not perceived as human beings*. *They [SIC*: *the public] think that they are ‘objects’ and they look at them like objects*” (psychologist, respondent 12). A driver for a social service organization stated: “*These children are already labeled*. *For people*, *these are the children who spoil everything on the street*. *They are not considered as children*, *because they do not grow up in a family*” (respondent 2). The cycle of labeling, stereotyping and social exclusion is illustrated in another statement from a social worker:

They are excluded… ‘typical’ children do not accept street-connected children. They do not like them; they are afraid of these children. They are afraid that street-connected children will steal something from them, will harm them… their parents [parents of YWLS] are afraid of ‘damaging’ their children by contact with street-connected children. Neighbors do not like them, as well, these children are labeled as so-called, the ‘street children,’ the criminal, the thief… and there are millions of labels for them, and these children constantly have to justify that they are not.

(respondent 18)

In subtheme 1, study participants detailed how public stigma manifested within different public institutions to exclude youth from accessing services and entitlements. Across interviews, stigma was reported to be common in schools. A psychologist described the different stigmatizing beliefs they encountered from teachers and educational administrators when trying to enroll YWLS in school: “*street-connected children are different*, *[they are] unable to study*, *and they can only bring problems to school*” (respondent 12). Another psychologist explained that some school administrators were reluctant to enroll YWLS because the mere presence of YWLS was thought to ‘spoil’ the school: “*The situation is very difficult at schools*. *When we take children from the center and engage them in activities at school*, *most teachers and school administrators are not tolerant towards these children…They say*, *I don’t want to ruin my school*” (respondent 20).

Across interviews, providers reported that schools were often hostile places for YWLS, with many being bullied by other youth, or experiencing hostility from administrators, teachers and parents of other youth. A manager (respondent 11) reported that YWLS have “*negative emotional experiences at school*” and described how other children use their association with the social service organization to make negative inferences about YWLS: “*Some of the children who receive services from the center go to school*. *There*, *they meet and interact with typical children*. *These children are very mean to them and say ‘Pah*, *what do you want there*? *Why do you go to that center*? *It means you have problems*. *It means you are a bad child*.’” A social worker described how the negative relationships with peers affected children’s ability to make friends with same-aged peers: “*They do not have school friends*. *They do not want to make friends at school because they are very discriminated against at school*” (respondent 18). While not all teachers were described as stigmatizing YWLS, a psychologist participant noted that some administrators treat their families poorly: “*There are people at school who are not aggressive towards the children*, *but they are aggressive towards their parents*…” (respondent 20).

In subtheme 2, a number of respondents detailed the different their efforts to overcome teachers and school administrators who attempted to avoid enrolling YWLS in schools. A social worker described how they had to invoke the law or connections with governments officials in order to enroll a female beneficiary in school:

Here’s an example, last year I tried to enroll a girl in one of the public schools. The school refused to enroll the girl. The reason the school refused was that they didn’t have a place in the class. I warned them that it was against the law not to accept the child. Then they said that they had no desk, I told them we would get a desk, we would either buy it ourselves or we would make it in the workshop… I finally called a representative of the Ministry of Education. After that, the director of school agreed to add a place for her, and the child joined the school.

(respondent 16)

Another social worker described how they also relied on the legal structure to overcome a school administrator’s initial refusal to enroll their beneficiary in the school: “*The director informed me that the school did not have places and could not enroll the child in the school*. *Unfortunately*, *it was a lie…*. *I talked to a teacher I knew*, *and she told me the opposite*: *that she had a place…I involved the Public Defender’s Office and after that…he had no other choice … and enrolled the child*” (respondent 5).

### Theme 2. Dirty and despicable young criminals: Intersectional stigma for ethnic minority YWLS

Across the interviews, key informants discussed their perception that youth of Romani and Kurdish-Azeri ethnic origins were subjected to particularly strong levels of public stigma. When describing the many labels used to denigrate YWLS, a manager of a social service organization noted that people used stigmatizing language and called for violent actions against ethnic minority youth:

I won’t say it all, of course, but very often people say about these children: “They shouldn’t be saved, but they should be killed;” “They are young criminals,” “They are despicable,” “They are dirty,”…Particularly, people talk like that for ethnic minorities…

(respondent 19)

A psychologist noted that stigmatizing language and actions also translated to how the public perceived deservingness of social services, with people not wanting to call government-sponsored service hotlines if they perceived youth as being of an ethnic minority origin:

People have especially bad feelings towards Kurdish children and Roma children. They call them ‘zigan.’ If they think that a street kid is Georgian and begging in the street without parental supervision, they will call the hotline. They say, in these words: ‘*They are Georgians*, *not zigan*’ [Roma] … They think that a Georgian child should be taken care of, but if she/he is zigan, it is not a problem.

(respondent 4)

A peer educator with a former history of living and/or working on the street also described the hostile attitudes and actions of the public towards Azeri-Kurdish youth: “*Seventy to eighty percent of mainstream society has very bad attitudes towards Azeri-Kurdish children*. *People scream at them; they say curse words…If a street kid sits next to them*, *people’s facial expression shows that they are disgusting*” (respondent 10).

In subtheme 3, providers described their experiences with ethnic minority youth being excluded from schools and healthcare settings. Respondents described how Azeri-Kurdish and Romani youth were bullied for having surnames that were easily identifiable as being ethnically non-Georgian. For example, a manager (respondent 13) narrated their experiences when enrolling Georgian and ethnic minority youth in school:

It’s easier for Georgians to integrate into the school because there is no language barrier and because they aren’t the victims of bullying. Their surnames, their names, and the way they look—it’s noticeable that they are not ethnically Georgian…. I remember how difficult it was to integrate two Azerbaijan-Kurdish children into a school. When I wanted to introduce the brother of one child, who was Azerbaijan-Kurdish and had a very different name, all of the other children laughed at him.

A psychologist (respondent 12) also described the difficulties with enrolling and keeping Azeri-Kurdish children in schools:

Perhaps 2% of Azerbaijan Kurdish children who are with us are enrolled in schools by our program, though they go to school less…. They were going to school for a short period of time, and then they got bored after a while, and unfortunately, society did not accept them….. these stereotypes and approaches are so strong at schools, that’s why children refuse to attend school. In fact, Azerbaijan-Kurdish children do not attend school.

Two providers also described encountering discrimination towards ethnic minority YWLS in health care settings. For example, the manager who described the difficulties with enrolling the two brothers in school (respondent 13) described how a doctor refused to provide care for an ethnic minority child under the guise of not having time: “*I brought an Azerbaijan-Kurdish child to the hospital … they made this child wait for many hours… when I complained*, *the doctor said that he did not have time…*. *This is very common*. *They prefer clean children with light skin*.” Similarly, a psychologist (respondent 12) described trying to have one of their beneficiaries treated for scabies, again noting how the doctor used time as an excuse for refusing to provide medical treatment:

I took a Kurdish beneficiary to the hospital…I told the doctor that I accompanied him, and the doctor looked at me with a surprised facial expression. “I have not examined him yet, I have not had the time.” What does that mean, “You didn’t have time?” The doctor prioritizes, for example, a patient with a lighter skin color.

Providers did not report instances of elevated stigma towards YWLS who are from Russia or other European countries, who also are part of the population of YWLS in Georgia [4, 68].

### Theme 3. Provider stereotypes about ethnic minority youth: Labeling, stereotyping and separating “us” from “them”

Despite the keen sensitivity that many providers displayed towards the stigma directed at ethnic minority YWLS, some of the providers also seemed to express negative beliefs and stereotypes about ethnic minority youth and their families. Negative views of ethnic minorities were endorsed by professionally trained social workers and psychologists, as well as by staff with a shared history of living and/or working on the street. For instance, a peer educator (respondent 10) contrasted the mental health of both groups: “*Ethnic minorities—Roma and Azeri-Kurdish—have more mental problems compared to Georgians*. *I have never met a Georgian street kid who had mental problems…*” When asked to elaborate on their rationale, they stated a belief that intellectual or developmental disabilities were more common among ethnic minority youth because they grew up in dangerous family environments:

For Roma children, mental retardation is more common among them because they grow up on the street. Imagine, we gave a remote control car to a Roma child, but he didn’t know what it was. But this is a simple thing: you tap it and it will go. Do you know, he couldn’t understand what it was. Mental retardation is more common among Azerbaijan-Kurdish children because they grow up actively on the street, they are born on the street… they walk with bare feet on broken glass. That is, they grow up in an environment where they cannot develop and learn. They have the same life. They go outside in the morning and come home and fall asleep in the evening. They can’t eat, they don’t know how to wash their hands if we do not help…. there is one child also in the street, who is a Kurdish child and when you ask something, it takes a long time for him to answer your question. I don’t know what to call it, what kind of diagnosis, but generally, it is a mental retardation because they grow up in an environment where they cannot learn

(respondent 10).

We also observed that providers used language about traditional families or normative child development to characterize where, how, and why they believed that ethnic minority youth were different from Georgian youth. For example, when describing the challenges encountered by ethnic minority youth, one social worker stated: “*Most of the ethnic minority’s families are not strong units… young women raise their children without fathers*” (respondent 16). This same social worker narrated how patriarchal structures within ethnic minority families led to reduced opportunities for Romani girls: “*Ethnic minorities have more patriarchal family structures… Girls are not allowed to stay single…Girls under 18 years old get married several times*. *For instance*, *a Roma woman can have five husbands in her life*.*”* This sentiment was echoed by a psychologist, who stated:

It is very common that Azeri-Kurdish and Roma girls of 13 start their sexual lives. It is mandatory to marry, have children and beg in the streets with children…. Their communities do not consider child marriages as criminal activity and a violation of a child’s rights

(respondent 12).

In subtheme 4, we identified negative stereotypes about childbearing in ethnic minority families and the sexual behavior of ethnic minority YWLS. For instance, a psychologist stated: *“More children*, *more money; this is the only reason for family formation in ethnic minority families*” (respondent 20). This belief was repeated by a social worker, who stated: “*For Azeri-Kurdish women*, *children are a source of income*. *As they have more children*, *they are perceived as being more vulnerable by society*, *and they have more opportunities to get more support”* (respondent 9). In another interview, a manager stated: “*A new baby for an ethnic minority means a baby with a new income*” (respondent 19).

Some providers also expressed stigmatizing beliefs about the sexual behavior of ethnic minority YWLS. In response to questions about sexual exploitation among YWLS, a psychologist reported that sex work was more common among non-Georgian youth: “*13–14 years old Azeri-Kurdish girls often visit Turkish night bars and talk to old men*, *drink and entertain themselves*” (respondent 12). Echoing this statement, another psychologist noted that non-Georgian youth were more likely to participate in anal sex than were Georgian youth: “*Anal contacts are very often with non-Georgian Ethnicities*” (respondent 3).

In the fifth and final subtheme, we found that some providers did not stigmatize ethnic minority YWLS, instead situating young people’s development within broader social contexts and structures. For example, a psychologist stated that social and linguistic exclusion could account for different developmental pathways between YWLS: “*Georgians are more developed than Azeri-Kurdish and Roma children because they have more information*, *as they speak Georgian*” (respondent 4). A social worker also noted that “*Ethnic minorities have more stable*, *regular partners than children of Georgian nationalities*” (respondent 11). A psychologist (respondent 20) also stated that while ethnicity was not related to engaging in commercial sex work, they believe that it shaped who had access to legal and social protections:

Ethnicity does not influence which groups of street-connected youth become involved in commercial sex, though it is still very rare for a Kurdish teenager to be involved in commercial sex. It’s very rare…. but ethnicity doesn’t define who becomes involved in the “ancient profession.” Ethnicity doesn’t determine this course of action; ethnicity can protect but could not stop involvement in sex work.

Finally, a manager described having favorable views of ethnic minority families, describing their networks as consisting of “*closely-knit family structures…*.*in which almost all members are relatives and very often live in one neighborhood*” (respondent 2).

### Theme 4. Youth fear, anxiety, and distrust: Provider perspectives on how public stigma affects YWLS

Almost all providers described their perceptions of how public stigma negatively impacts YWLS. Providers perceived that youth are deeply attuned to public stigma, “*when the child passes by them and you hate him*, *the child always feels it*” (manager, respondent 15). A social worker (respondent 18) described their belief that negative and violent interactions with the public lead YWLS to distrust not only social services, but also society at large.

They perceive some people as enemies… as they do not receive any social support,…. these children are called thieves and society is not friendly to them. It is natural that these children do not trust society, as society kicks the child out and society is not friendly to the child. It is natural that the child does not have healthy relationships with them, the child permanently thinks that he/she will be treated badly.

Another social worker stated: “*They fear how other people will react to them… They worry and they are nervous*, *they are afraid of other people and their environment*” (respondent 16). A psychologist (respondent 22) also described their thoughts on how negative experiences with the public traumatize YWLS and make it difficult to engage youth in services:

But sometimes, there is a child who does not want anything… he/she has nothing to do with you, does not want help from you. He/she is completely locked in and so frightened by the public’s experience that he/she does not want any help… any contact with us.

A peer educator noted that the distrust towards social services also was present among ethnic minority youth, who they believed would feign linguistic ignorance to manage their fear and distrust: “*Azerbaijan-Kurdish children know Georgian*, *but if you speak to them in Georgian*, *you may not be answered just because they are afraid of you*” (respondent 9). This belief was repeated by a social worker, who described the particular fears that ethnic minority YWLS encounter when navigating health and social services:

They have fears in a generally foreign environment, no matter where we take them, when they don’t know where we are going or how to behave in this case. They are afraid of going to the doctor. In particular, they have a great deal of fear, when they have a pain or something and we have to go to the hospital.. “I will escape from there, I will be killed there. I will be…” They are afraid of going to the doctor because they do not know what will happen there.

(respondent 17)

In addition to creating distrust and fear, providers described their perceptions of how public stigma led many youth to develop self-stigma. For many YWLS, self-stigma was said to manifest as feelings of shame, negative self-worth, and a heightened sense of being different from other youth. For example, a manager stated: “*They are ashamed because they think they are behind*, *as they never went to school*. *They are different than other children and it affects everything”* (respondent 19). A social worker elaborated on this phenomenon:

Once there was a celebration and the whole class was sitting, but the children didn’t eat, because they thought the whole class would watch them and laugh at them. It is their expectation…In fact, no one was looking at them, all of them were looking at their own dishes, but they have a moment where they fear that they will be watched, looked at and criticized by others. And what is important—this is also a moment of self-esteem…you imagine yourself differently…I think they are afraid of being laughed at, “If I behave in such a way, they will laugh at me.”

(respondent 5)

The same belief that YWLS came to understand themself as different from other young people was echoed by a manager, who described how many of their beneficiaries struggle with self-esteem: “*Yes*, *they have very low self-esteem*, *and one child told me*: *‘Do you know*, *today*, *everyone was looking at me*? *They were looking at how I was dressed when we went to the sport hall*” (respondent 11).

### Theme 5. Difficult working environments: Provider encounters with courtesy stigma

Providers described a range of negative interactions with the public surrounding their work with YWLS. For example, a psychologist described the negative looks they received when accompanying young people on the street: “*When I accompany a child*, *when we go together*, *I notice the gazes of other people*, *they may not be able to express what they want to*, *but I can see their attitude towards these children with my own eyes*” (respondent 4). This same participant described being met with “aggression” from people when attempting to defend children from negative comments or actions. A similar pattern of public disdain was described by other study participants, who characterized these interactions as a major job stressor: *“The society is very aggressive towards them… If people see me following these children*, *they give me such a bad look*, *so it is really overwhelming to do my job”* (psychologist, respondent 4).

Many study participants described being subjected to public rebuke and harassment when their connection to YWLS was visible to the broader public. Here, a manager describes the response they received when demonstrating affection towards one of their beneficiaries in public:

Once, when I was at the metro station, I met my beneficiary, and I gave her a hug. All the passengers in the wagon looked at me with disgust. They said to me, “You are the same. How can you kiss and touch this child and show affection towards her? What kind of woman are you?”

(respondent 11)

Another manager (respondent 19) described the negative comments they received when accompanying ethnic minority YWLS:

I always get negative comments from people when I am engaging ethnic minority children. I hear such comments: you should not give shelter to him, rather you should kill him, they are criminals, they are disgusting, they are dirty, and etc.

## Discussion

The present study examined how social service providers in the Republic of Georgia perceive the social contexts surrounding their work with YWLS, a population that has grown considerably in the past decade [4, 11]. Consistent with prior research in Georgia [4, 11] and other low- and middle income countries [2, 6, 69], we find that providers perceive that YWLS are a highly stigmatized group. Across interviews, providers detailed the ways in which they believe the broader public views YWLS as having a spoiled and tainted identity. The processes they described follow those detailed by Goffman [14, 15], who elucidated how processes of labeling, stereotyping, separation and discrimination can follow the ascription of a stigmatized identity. Indeed, providers described how the mark of being a “street child,” visibly ascertained through a young person’s appearance, physical location on the street, connection to a social service organization, engagement in begging or other street economies, or through presumed ethnic identification, is used to justify an array of negative beliefs, hostile attitudes, stereotypes, and discriminatory actions.

Institutional stigma [44] was reported to be common in schools. Multiple respondents detailed their perceptions of the ways in which teachers, administrators, students and their parents exhibited negative attitudes towards YWLS. In addition, providers tasked with enrolling youth in schools, such as social workers and social service managers, described instances when YWLS were denied access to education. In these cases, providers described how they relied on legal systems to successfully enroll youth. Additional research is needed to identify how best to address institutional stigma within public schools. While our data indicate that providers work to ensure that individual schools enroll eligible youth, this is but one step in addressing stigma towards YWLS. According to providers, the hostile attitudes directed towards YWLS can also lead some youth to distrust the social institutions tasked with serving them. In addition to disincentivizing engagement in education and social services, providers report that this may lead to self-stigma among YWLS [15]. In prior research with YWLS, self-stigma has been correlated with poor emotional health [1, 2, 70]. Future research should examine the role of self-stigma on young people’s mental health, with a particular focus on how self-stigma may vary between YWLS of different ethnic groups, as providers detailed a number of instances where institutions attempted to deny access to services like education and medical care to ethnic minority youth.

Across Europe and other former Soviet republics, there are well-documented histories of cultural racism, stigma, and discrimination directed towards Roma communities, i.e., anti-Ziganism and Romaphobia [49, 50, 52, 53, 58]. Several studies also have described anti-immigrant sentiment directed towards immigrants and non-native Georgians in Georgia [46, 47, 71, 72]. In the present study, providers perceived that ethnic minority YWLS experienced the strongest levels of public stigma, describing how the public uses particularly hostile and violent language, e.g., “*you should kill them*.” Providers also detailed instances where they believed that ethnic minority children were denied access to medical care based on the color of their skin. Similar to work documenting the presence of intersectional stigma among multiply marginalized groups [21, 28], analysis of provider responses indicates that ethnic minority YWLS in Georgia may experience intersectional stigma from being connected to the street *and* the stigma of being of Roma or Azeri-Kurdish origin.

The social exclusion of Roma communities is not unique to Georgia and has been well documented in other countries that are home to Roma children, families, and communities [54, 56, 58, 73–76]. For example, examining processes of social exclusion among Roma girls and women in Romania, Greece, and the Czech Republic, Martinidis et al. [56] found that Roma children were regularly discriminated against in schools, including being denied enrollment, segregated from non-Roma children, bullied, and unnecessarily placed in special needs settings. A recent systematic review also found that Roma groups experienced stigma and discrimination in healthcare settings [76]. To date, less research has explored this phenomenon among Azeri-Kurdish families in Georgia [50]. Our findings suggest that additional research on stigma towards Azeri-Kurdish YWLS is needed.

Although providers recognized stigma as a negative animating force in the lives of YWLS, especially those from ethnic minority groups, some providers also reported negative stereotypes about ethnic minority youth. In their responses, we observed that providers invoked oft-repeated stereotypes about Roma communities, refugees, and immigrants, such as the belief that ethnic minority families have children solely to access financial resources [52], that ethnic minority youth are mentally, socially and developmentally inferior due to familial or cultural characteristics [52, 56], or that ethnic minority youth engage in stigmatized sexual behaviors [52, 55]. A number of scholars [52, 55, 77, 78] have documented the processes through which media outlets, politicians, social service providers, and human rights organizations “discursively construct Roma childhoods as dangerous and devoid of the resources that lead to healthy adulthoods” [52]. While being connected to the street and/or living in a violent family both increase vulnerability to poor social and health outcomes—irrespective of ethnic group membership—numerous studies have documented the powerful roles of stigma, discrimination and social exclusion for Roma groups [53, 56, 57, 58]. Similarly, poverty and low education—two potential indicators of social exclusion—are well documented correlates of child marriage among Roma and other groups [79–82]. It is notable, then, that some providers defaulted to stereotypes and cultural deficit narratives to explain their observations of child marriage or transactional sex among Roma and Azeri-Kurdish YWLS. These narratives also contrast with those providers who discussed the role that stigma, language barriers, or social exclusion may play in their work with ethnic minority YWLS.

We also observed the expression of negative stereotypes about ethnic minority YWLS from study participants with advanced educational degrees, e.g., social workers and psychologists, as well as peer educators—young people with their own history of working and/or living on the street. Prior work examining stigma towards Roma peoples has found that providers can research has found that provider stigma can persist in the face of education [40, 41], pointing to the need for multi-component approaches to addressing stigma. Future research should examine the extent to which different types of providers who report stigmatizing beliefs about ethnic minority YWLS enact actual stigma against these same youth, as prior research indicates that provider stigma can manifest as institutional stigma [50, 51]. Given the important role that social service providers play in supporting YWLS, future research should examine the factors that account for different stigmatizing beliefs among providers, as well as the factors that allow some providers to understand ethnic minority YWLS through broader social, cultural, and political processes.

This study also adds to the growing body of literature on the role of courtesy stigma [32–34], documenting that providers working with YWLS report being subjected to public stigma through virtue of their professional role and association with youth on the street. Respondent 11’s example of hugging a young person on the street and being asked “*How can you touch this child*? *What kind of woman are you*?,” illustrates the concept of courtesy stigma. The child in question is so stigmatized that the act of extending a hug to her elicits a strong public rebuke, where the provider’s moral character is questioned as her identity is rendered equally suspect and tainted. Most of the providers who described processes of courtesy stigma indicated that it created an overall stressful work environment. Similar to self-stigma, courtesy stigma is related to reduced psychological well-being [83] and prior research has found that courtesy stigma is differentially experienced by frontline workers [32]. We were not able to explore the factors that shape how providers conceptualize and respond to courtesy stigma; future research should examine the different strategies that providers invoke to manage the potential impact of courtesy stigma on their personal and professional well-being.

### Limitations and future research

The present study is not without limitations. First, our data do not include the experiences of the many YWLS in Georgia, whose voices are critical for understanding how stigma shapes their lives. Although all members of the research team had histories of serving YWLS in Georgia and the United States, no one had a history of living and/or working on the street themselves and no current YWLS were members of the research team. Including YWLS on the research team could have yielded additional insights on our positionality as outsiders to street-life in Georgia. As future research moves forward, it may be helpful to utilize youth participatory action research (YPAR) methods to partner with Georgian and ethnic-minority YWLS as equal partners in the research endeavor. Prior research has found that participatory methods with YWLS can develop trust, counter stigma, and foster social inclusion.[84–86] In addition, ethnographic research that includes participant observation in both social service settings and street-based outreach contexts may be particularly helpful for illuminating the complex social, relational, and organizational dimensions of stigma [50] that are experienced by diverse groups of YWLS.

Second, the data are based on a convenience sample of providers from social service organizations in two major urban centers in Georgia. Their views may not represent the perspectives and experiences of providers in other areas of the country or outside of Georgia. Nevertheless, we interviewed a diverse array of providers, obtaining rich perspectives on stigma from different types of professionals serving YWLS. To date, we know of no peer-reviewed interventions addressing stigma towards YWLS in Georgia. Our data suggest that this work is necessary, and that interventions must take an intersectional approach to reduce stigma at the provider, institutional and public levels. As this work proceeds, it may be helpful to learn from the global literature examining anti-stigma interventions for mental illness [87–91]. YPAR methods will be especially important to use in future intervention research, as this will position youth as agentic actors who can and should be shaping the practices, programs and policies aimed at their health and well-being [85]. These data are from a cross-sectional study; as such, no casual mechanisms can be determined. Future research can build upon these findings by using longitudinal designs to explore the impacts of stigma on young people’s biopsychosocial outcomes and how stigma processes and outcomes may change over time.

Despite these limitations, this research offers important insights into how providers perceive the different types of stigma directed towards diverse groups of YWLS. Study findings point to the need for additional research with providers and young people, both to support providers to manage the public stigma directed at YWLS, and to support youth who work and/or live on the street to manage the stigma directed at them, particularly those from ethnic minority communities.
