# Supplementary material for: “How can you kiss and touch this child and show affection towards her? What kind of woman are you?”: Provider perspectives on stigma towards native and ethnic minority street-connected youth in the Republic of Georgia
Source: PLoS One. 2023 Jun 2;18(6):e0286710. doi: 10.1371/journal.pone.0286710 (PMC10237414; doi:10.1371/journal.pone.0286710)
Supplement: Attachment — Submitted filename: Response to Reviewers sh.docx [file pone.0286710.s001.docx]

September 24, 2022

Thomas E. Guadamuz, Ph.D., M.H.S.
Academic Editor, PLOS ONE

PONE-D-22-13919

“They walk with bare feet on broken glass, they are not developing normally, and they are not learning anything”: Provider perspectives on stigma towards native and ethnic minority street-connected youth in the Republic of Georgia

Dear Dr. Guadamuz,

We have appended our revision for PONE-D-22-13919. We appreciate the comments from the Reviewers and the opportunity to revise the manuscript. Below, please find our responses to journal-specific requests, as well as our specific comments and rebuttals to Reviewer comments. As requested, we have appended a marked up copy titled 'Revised Manuscript with Track Changes' and an unmarked version titled 'Manuscript'.

Below are our responses to journal requirements.

1. Please ensure that your manuscript meets PLOS ONE's style requirements, including those for file naming.

The manuscript has been revised to meet PLOS ONE’s style requirements.

2. In your Data Availability statement, you have not specified where the minimal data set underlying the results described in your manuscript can be found.

You can find minimal data set on the Ilia State University repository.

http://eprints.iliauni.edu.ge/cgi/users/home?screen=EPrint%3A%3AView&eprintid=11133

3. As noted above, we uploaded a minimal data set to:

http://eprints.iliauni.edu.ge/cgi/users/home?screen=EPrint%3A%3AView&eprintid=11133

4. Please include your full ethics statement in the ‘Methods’ section of your manuscript file.

We have added the full name of the IRB ethics committee who approved the study to page 8. It now states: “All study protocols were approved by the Ilia State University Ethics Committee on June 26, 2018 (R/333-18).” In the revised submission, the statement regarding written informed consent is on page 8.

5. Thank you for stating the following in the Acknowledgments Section of your manuscript: “This research was funded by the Shota Rustaveli National Science Foundation. (SRNSFG) [#FR 17_31]”. We note that you have provided additional information within the Acknowledgements Section that is not currently declared in your Funding Statement. Please note that funding information should not appear in the Acknowledgments section or other areas of your manuscript. We will only publish funding information present in the Funding Statement section of the online submission form. Please remove any funding-related text from the manuscript and let us know how you would like to update your Funding Statement.

We removed funding information text from the manuscript. However, we need to include this statement in the Funding Statement Section:

“This research was funded by the Shota Rustaveli National Science Foundation. (SRNSFG) [#FR 17_31]

Principal Investigator: Shorena Sadzaglishvili”

Please change the online submission form according to this information.

6. As requested, we now clarify the role of the funding agency in the study. The amended Role of Funder statement is below:

“The funders had no role in study design, data collection and analysis, decision to publish, or preparation of the manuscript.”

7. As requested, we clarify the Competing Interests below and have updated the online submission form:

“The authors have declared that no competing interests exist.”

8. We reviewed the reference list, as requested. No references were deleted and no references are based on retracted papers. We did, however, add references in response to requests from Reviewer 1. Specifically, references 76 to 87 are new.

**Reviewer Comments**

**Reviewer 1 provider several suggestions to strengthen the literature, methods, and analytic interpretations. Below, we summarize and respond to Reviewer One’s specific comments.**

1. Reviewer 1 believed that our data on provider stigma towards ethnic minority YWLS was too thin and that it wasn’t clear enough to judge that what KIs said about YWLS was stigma perception or knowledge from frontline experiences working on the street. The Reviewer concluded that there was little evidence to support the claim on par 2 page 10 that “we found that providers themselves report high levels of stigma toward ethnic minority YWLSW.”

We appreciate the opportunity to clarify the nature and strength of the findings that a subset of providers demonstrated stigma towards ethnic minority YWLS, as opposed to repeating stigmatizing attitudes and beliefs held by others. On page 10 of the revised manuscript, we have edited the introduction to the results to state:

“First, we found that providers described high levels of public stigma directed towards YWLS, with public stigma manifesting as institutional stigma to limit youth access to key services like schools and medical care. Second, we found that cultural racism [57] produces intersectional stigma [21,47] for Roma and Azeri-Kurdish youth, who encounter stigma based on their status as street-children and as ethnic minorities. In addition, we found that a subset of providers who discussed public stigma towards YWLS also reported negative attitudes and beliefs about ethnic minority YWLS, illustrating how cultural racism may manifest as provider stigma for multiply marginalized YWLS. Providers described how high levels of public stigma negatively impact YWLS, who often fear and distrust large segments of Georgian society. Finally, we found that providers who serve YWLS describe encountering courtesy stigma, which creates a stressful service delivery context. Each theme is discussed below, with illustrative quotes from interviews along with the professional role of each respondent.”

To further clarify how the data supports our claim, we have reorganized the results section that describes provider stigma towards ethnic-minority youth (see pages 14-17). In the revised results, we now lead with the data describing how providers contrasted Georgian youth with ethnic minority youth, noting how the comparison reveals a difference in attitudes and beliefs between the two groups of YWLS. In addition, we have more clearly noted that these beliefs were expressed among a subset of providers and not all providers. We believe that these changes address the Reviewer’s concerns and allow us to clarify the findings.

2. The Reviewer posed several methodological questions. First, they asked us to reconsider using the term semi-structured in-depth interview; here, the Reviewer thought that one could not use in-depth probes if the interview question was structured. They also noted quantitative data would be expected if a semi-structured form was used. In addition, the Reviewer wanted additional information on how the 22 key informants were included in the sample, i.e., where the recruited officially or unofficially, accidental, or through network relationships?

We used a semi-structured interview guide for the key informant interviews. The questions were developed by the Research Team based on the extant literature and stakeholder feedback and designed to elicit provider perspectives on: (1) the social network characteristics of YWLS, (2) youth’s involvement in substance use and sexual behaviors that may heighten the likelihood for HIV transmission, and (3) the social contexts of youth engagement and service delivery. During the interviewers, data collectors posed questions to each key informant and used probes to elicit additional details as needed. Semi-structured interview guides are ideal for qualitative research as they provide structure and flexibility. As opposed to a structured guide, where questions may be close-ended, questions in a semi-structured are typically open-ended. The open-ended nature of the questions allowed us to elicit thicker and more nuanced descriptions from key informants and to then analyze these data for emergent themes across interviews. On page 8 of the revised manuscript, we note that we used open-ended questions and that interviewers used probes to elicit additional details from providers.

Although quantitative approaches can be used to analyze qualitative data, this was not the purpose of the present study. Rather, we sought to qualitatively explore how providers describe their work with street connected youth. As such, we did not apply a quantitative approach to analyzing the data.

Finally, the informants were recruited from all existing social service organizations in two major cities, Tbilisi and Rustavi. We do not provide names of the organizations so as to ensure participant confidentiality. The key informants did not have an official relationship with the study and were not involved in developing the study or interpreting study results. We have added this information to page 8.

3. Reviewer 1 asked us to consider how including youth or adults with experiences of living and/or working on the street would have shaped our analysis, interpretation and any lessons learned.

We appreciate the Reviewer’s point and agree that including YWLS would have yielded additional insights on our role as outsiders. In the limitations section, we acknowledge this point and discuss the importance of involving youth with lived experience as members of the research team. In so doing, we note the need for youth participatory action research with YWLS in Georgia, which would include youth as full partners in the research endeavor. Here, we briefly note that prior research using YPAR with YWLS has yielded important benefits and is important for giving youth a voice in the practices, programs and policies that shape their lives (please see page 25).

4. Reviewer 1 noted that it would be useful to discuss who the providers were that expressed stigmatizing beliefs towards YWLS and to describe the factors that account for their beliefs, as well as what differentiates them from providers who did not express these beliefs.

In the original manuscript, we included the professional role of each provider, e.g., social worker, psychologist, coordinator, peer educator, etc. We did not provide additional details on the key informants, as this information would risk deductive disclosure and a potential loss of confidentiality for those providers who agreed to participate. In addition, because the interview guide was not designed to focus on stigma, we did not have follow up questions that elucidate how providers came to hold these beliefs and the factors that might differentiate between those who did and did not endorse stigmatizing beliefs towards YWLS. As such, we are not able to identify what factors account for the different levels of stigmatizing beliefs among providers. Nevertheless, we believe this work is necessary to move forward with programs to address provider stigma towards different groups YWLS. In the discussion section, we note the need for future research in this area (see page 23).

5. The Reviewer noted that while the paper addressed the knowledge gap on provider perspectives on stigma directed towards YWLS, it needed additional revision to fulfill the stated purpose. While they stated that the literature review was good, they requested that we add more relevant studies on provider perspectives on stigma towards YWLS.

To date, few studies have examined the perspectives of Georgian providers on stigma towards YWLS, a gap this study seeks to address. Reviewer 2 also asked us to condense the literature review section. Nevertheless, we added information on provider stigma towards YWLS, reporting details of a 2020 report from UNICEF about Georgian YWLS and a prior study with Georgian YWLS (see pages 5-6).

**Reviewer 2:** We were pleased that Reviewer 2 judged the paper as excellent and a pleasure to read. They made several minor comments and provided two suggestions to strengthen the publication, which we respond to below.

1. Reviewer 2 asked that we review the manuscript to ensure there were spaces between words and references and provided an illustrative example from the original submission.

We have edited the revised manuscript to ensure appropriate spacing (see page 1).

2. The Reviewer pointed out that we were missing the word ‘life” in the following sentence "Currently, social service delivery for YWLS in Georgia focuses on both preventing youth from entering street life and on reintegrating young people into mainstream social [4]."

We have added the missing word “life” to this sentence.

3. Reviewer 2 asked us to edit the introduction to more concise, which would help to engage a wider audience.

We have made considerable changes to the introduction. Specifically, we edited the introduction on YWLS in Georgia and literature review on stigma. In so doing, we have cut approximately one page from the introduction (please see pages 1-6).

4. Reviewer 2 requested additional information on how the interview guide was developed.

The interview guide was based on a literature review of studies and reports on YWLS in Georgia and with feedback from an advisory board that included community members involved with street connected youth. This information can be found on page 8.

5. The Reviewer requested that be more explicit in the results on how provider stigma towards ethnic minority YWLS is tied to the ways in which youth and their families are seen as deviating from traditional conceptions of family drawn from patriarchal norms.

We appreciate this suggestion and agree that patriarchy is likely an important factor that facilitates stigma towards ethnic minority YWLS and their families. Although we had briefly mentioned patriarchy in the prior version, we have elaborated on how provider’s statements reflect patriarchal values in in the revised results (see pages 14-17).

6. The Reviewer also asked us to elaborate on the role of patriarchy in facilitating provider stigma towards ethnic minority YWLS in the discussion section.

We have added a brief paragraph on the role of patriarchy in facilitating provider stigma towards ethnic minority YWLS in the discussion section. Here, we situate our results in the broader on literature elucidating patriarchal culture in Georgia (see page

7. Finally, Reviewer 2 noted that they were left wondering if we had thoughts on the types of interventions or approaches could be taken to reduce stigma.

We appreciate the Reviewer’s focus on interventions, as this work is part of a pilot study that we hope will lead to future interventions. At this time, we do not yet know what format or content should be targeted, as additional research is needed to identify the primary correlates of stigma amongst providers and the public that could serve as intervention targets. However, we believe that future interventions must be address intersectional stigma, target multiple social-ecological levels, and involve youth directly, ideally through youth participatory action research. We have mentioned this and the potential to learn from the global literature on mental illness stigma interventions on page 25 of the revised manuscript.

We thank you and the Reviewers for the comments and opportunity to revise the manuscript. We believe the revised manuscript is stronger and hope that it is now ready for publication in PLOS One.

Sincerely,

Shorena Sadzaglishvili, PhD, MSW

Professor of Social Work

Ilia State University
