# Supplementary material for: “How can you kiss and touch this child and show affection towards her? What kind of woman are you?”: Provider perspectives on stigma towards native and ethnic minority street-connected youth in the Republic of Georgia
Source: PLoS One. 2023 Jun 2;18(6):e0286710. doi: 10.1371/journal.pone.0286710 (PMC10237414; doi:10.1371/journal.pone.0286710)
Supplement: Attachment — Submitted filename: Response 2.20.23.docx [file pone.0286710.s002.docx]

February 17, 2023

PONE-D-22-13919R1

“They walk with bare feet on broken glass”: Provider perspectives on stigma towards native and ethnic minority street-connected youth in the Republic of Georgia

Dear Dr. Arije,

Thank you for the opportunity to further revise our manuscript. We appreciate that the original Reviewers determined that we had responded to their primary concerns. We also welcome the opportunity to further strengthen the purpose, findings, and relevance of the study. Below, please find our responses to your comments and questions. In most cases, we made the requested changes. In some cases, however, the requested change or clarification was already present in the manuscript; in order to avoid repetition, we did not add duplicate information to the revised manuscript but rather sought to clarify the requested information. As instructed, we have made all edits in Track Changes.

**Introduction**

1. The Editor asked to clarify the type of providers we interviewed and are referring to in the manuscript. They pointed out two specific passages and requested we clarify the type of providers early in the manuscript:

“Although some research has examined the social, health, and economic needs of YWLS in Georgia [4], few studies have explored how providers perceive, manage, and respond to the stigma directed towards their beneficiaries.

“In a qualitative study with providers serving sex workers, providers described encountering courtesy stigma from friends and family, other providers, and the broader public [32].”

In the revised manuscript, we have clarified that we are referring to social service providers who deliver services to YWLS in both the abstract and introduction. In addition, we have clarified the types of providers in the literature review. For example, for citation 32, this was health service providers that included psychologists, nurses, drug counselors and physicians (see page 5).

2. The Editor noted that the following sentence states the method and not the study objective: “The present study helps to address this gap by conducting in-depth interviews with 22 social service providers who serve YWLS in Tbilisi and Rustavi, two major cities home to an estimated 1,094 YWLS [11].”

In the revised manuscript, we have edited the sentence on page 2 to state: “The present study helps to address this gap by examining how social service providers who serve YWLS in Tbilisi and Rustavi, two major cities home to an estimated 1,094 YWLS [11], perceive the social contexts in which they deliver services to YWLS.”

3. The Editor requested that we clarify the types of managers described in the following sentence: “These groups are based in major urban centers and led by State Senior Social Workers, with allied health professionals like psychologists, managers, peer educators, and logistics officers.” On page 3 of the revised manuscript, we now state that we are referring to managers of social service organizations.
4. The Editor noted that it is not necessary to present subtitles in the introduction. As requested, we have deleted all subtitles from the introduction.
5. The Editor requested that we delete the following sentence, as it repeated a prior statement: “To date, little research has examined provider perspectives on the role of stigma in delivering services to diverse groups of YWLS in the Georgian context.” We have deleted this sentence.
6. The Editor noted that we conflated the study objective with study methods, as evidenced in the following sentence: “The present study helps to address these gaps by analyzing qualitative data from in-depth interviews conducted with key informants (n=22) employed in social service organizations working with YWLS in Tbilisi and Rustavi, Georgia.” They requested we consider replacing with this statement: “The aim of this study was to assess the perspectives of social service providers for YWLS on stigma towards native and ethnic minority street-connected youth in the Republic of Georgia.”

We appreciate the Reviewer’s point. However, the purpose of our study was not to explore stigma but to explore provider perspectives on the social contexts in which they serve YWLS. Themes related to stigma and the ways in which providers perceive differential stigma among native and ethnic minority youth emerged from the data. Nevertheless, we agree that we have conflated the study objectives with study methods and have amended the sentence to note that the objective of the study was to better understand how diverse social service providers perceive the social contexts in which they deliver services to street-connected youth (see page 7).

**Method**

7. The Editor noted: “This section should start with a statement about the study design, followed by the study settings/location. This can then be followed by descriptions of the study populations/participants.”

On page 8, we have now added a description of the study design to the first sentence of the Method section.

8. The Editor requested that we consider replacing this sentence “Key informants did not have an official relationship with the study and were not involved in developing the study or interpreting study results” with: “None of the study participants had official relationship with the study and were not involved in developing the study or interpreting its results.” This change has been made in the revised manuscript (see page 8).
9. The Editor requested we clarify our use of probes, based on the following sentence: “When appropriate, interviewers also used probes to elicit additional details from KIs” They noted that one should ask when is it not appropriate to use probes in open-ended interview sessions, as probing is a cardinal interviewing skill in qualitative research.

We agree that probing is a fundamental interviewing skill in qualitative research. However, when respondents provide thick descriptions that fully answer a question, it may not be necessary to use a probe. Rather, probes were selectively used to elicit information when responses were vague or unclear. Nevertheless, we have deleted this sentence so as to avoid any confusion on the part of readers.

10. The Editor asked us to please be specific about the language in which interviews were conducted and the process of translation if there was any. This information was included in the original submission: all interviews were conducted in Georgian (see page 9) and translation was conducted using the Euro-Reves method. We now briefly describe the translation method on page 10.
11. The Editor requested clarity about our use of the term cultural racism in our Positionality section, where we stated: “This process was especially useful when talking about the presence of cultural racism directed towards ethnic minorities in Georgia, as this manifestation of stigma is both similar and distinct from racism within the United States.” The Editor noted that the term racism was being introduced for the first time in the manuscript but there was no indication in the introduction that this study was directed at issues about racism.

The study was not directed at racism but rather at understanding how social service providers understand the social context of service delivery to YWLS. Through the analysis of the qualitative data, we identified the broad theme stigma. Within stigma, we then identified sub-themes, including the ways in which providers report their perceptions of stigma towards ethnic minority YWLS. We then drew on a large body of research that has documented cultural racism towards Roma groups. In the revised manuscript, we have attempted to address the Editor’s concerns in several ways. First, in the introduction to the manuscript, we have expanded our discussion on the specific forms of stigma and discrimination experienced by Romani groups (see page 6). Whereas the prior submission briefly mentioned cultural racism, we now define both antiziganism and Romaphobia, drawing on literature that conceptualizes these phenomena as forms of cultural racism that are connected to structural stigma. We believe these changes better prepare readers for our mention of cultural racism in the positionality section.

**Results**

12. Although the Editor noted that this was a stylistic preference, they felt that the summary of the themes we presented in the results was not appropriately placed. They noted that some authors place such a summary in their data analysis section and asked that we rework this particular paragraph into the summary of the whole work.

We agree with the Editor that this is a stylistic point. Nevertheless, we have attempted to be responsive to the Editor’s comment by placing the summary of the results in the data analysis section. It now proceeds the positionality section. In addition, we have ensured that the summary follows the order of the results. Please see page 10.

13. The Editor stated: “The claim of cultural racism is not immediately justifiable as presented because there is no indication of the ethnicity of the provider in the immediate context, unless the authors can clearly state that there are no ethnic minorities among the providers, which doesn’t seem to be the case.”

The Editor rightly notes that the sample was not exclusively Georgian, with a small number of respondents identifying as Azeri-Kurdish or Romani. However, we do not include the ethnicity of providers because doing so would jeopardize the anonymity and confidentiality of the providers, who could be deductively identified by providing both ethnicity and professional role given the overall small sample and community of local organizations and providers working to support YWLS. We politely disagree with the Editor that it is not possible to infer some level of cultural racism from the data. First, some providers described their beliefs that ethnic minority youth are subjected to negative attitudes or discrimination based on their perceived ethnic membership or on the color their skin. In addition, there is a large volume of prior research documenting high levels of anti-ziganism directed towards Romani groups, much of which is situated within the context of cultural racism. Thus, the statements from providers about ethnic minority youth are not without context. In response to the Editor’s concerns, we have expanded our discussion of anti-ziganism and Romaphobia in the introduction (page 6). We also have included additional quotes from providers, where they compare and contrast different groups of youth. In the results and discussion, we have ensured that none of our statements are causal and that providers are reporting their perceptions. In the results, we now title the section Intersectional stigma and have deleted the phrase about cultural racism producing intersectional stigma (see page 14).

14. The Editor requested that we not use the term subset in the following sentence: “Finally, we found that a subset of providers who discussed public stigma towards YWLS reported negative attitudes and beliefs about ethnic minority.” The Editor then stated: “How many were in this subset of providers? For instance, can authors say how many of the 22 persons interviewed had this attitude, and describe commonalities among this group. While frequency is not a focus in qualitative research, it becomes significant when there are weighty claims. Authors should demonstrate that this is not a chance finding and that there are some commonalities among those who seemed to express racism.”

The Editor rightly notes that there are different perspectives on whether qualitative results should be quantified. We agree and have attempted to demonstrate the strength of our findings by listing the relevant quotes under the identified themes, the professional role of the respondent, and respondent number. As requested, we have removed the use of the word subset. We also have provided additional data on the ways in which providers discussed ethnic minority youth. We do not believe that the finding is a chance one, as providers did not tend to describe Georgian or other YWLS with similar language; we have moved that statement from discussion to page 16 of the results.

15. The Editor did not think we were being transparent enough with this statement: “Notably, provider stigma towards ethnic minority YWLS was observed among a number of staff with advanced educational degrees, e.g., social workers and psychologists, as well as peer educators—young people with their own history of working and/or living on the street. Prior research has found that provider stigma can persist in the face of education [40,41]”

This section was included in the discussion because we believed it was an interpretive point that was better suited for the discussion versus the results, a point raised by the Editor in another comment. In the revised manuscript, we now note that we included respondent role and study identification number as part of our coding (see page 10).

16. The Editor requested that we modify the following sentence on page 12: “Analysis of provider data revealed that YWLS are viewed as having a “spoiled identity,” from which labeling, stereotyping, separation and discrimination follows [14,15].” They noted that it should be a discussion point, not a result from this study. This has been deleted from page 12 and is now included in the discussion on page 22.
17. The Editor noted an erroneous return between the sentence after: “Providers also detailed how public stigma manifested within different public institutions to exclude youth from accessing a range of services and entitlements.” This change has been made (see page 12).
18. The Editor requested we move away from quantitative language in the following sentence: “Institutional stigma was especially prevalent in schools,” and use a more qualitative term, such as “Stigma was reported to be common in schools.” This change has been made on page 12 of the revised manuscript and we have edited the manuscript to ensure this language is not present in other places.
19. As a general comment, the Editor requested that we report what the interviewees said rather than our own interpretation of what was said. As an example, they noted that institutional stigma is a technical term and if it wasn’t used by the study participants, then we should not use it in the result. They recommended we include it in the discussion section instead. We have organized our themes to map on to larger theoretical constructs, like public, institutional, intersectional or courtesy stigma and have edited the results to ensure that it does not read like providers are using these terms.
20. The Editor requested that we reconsider how we work quotations into narratives, noting that it didn’t read well both grammatically and stylistically. The Editor provided the following example: “Psychologist explained why school administrators are reluctant to enroll YWLS because their mere presence is thought to spoil the school: “...in most cases, there is less tolerance, and they think it's something: ‘I don't want to ruin my school.’” They requested the following edit: “A psychologist participant explained that some school administrators were reluctant to enroll YWLS saying, for instance, “…I don't want to ruin my school…,” because the mere presence of YWLS in the school was thought would ‘spoil’ the school.

This reflected a typographical error (i.e., missing A at the start of the sentence). We have edited this sentence but did not make the complete change as the quote was expanded to address another request from the Editor to provide additional data on the ways in which schools were hostile to YWLS (see Item 21 below). In addition, we have reviewed and edited quotes throughout the manuscript. Often times, this included stating the role of the study participant in the introduction to the quote, rather than in the parentheses.

21. The Editor requested that we provide additional quotes for the statement that “In addition to overt attempts to exclude YWLS from schools, providers noted that schools were often hostile places for YWLS, with many being bullied by other youth, or experiencing hostility from administrators, teachers and parents of other youth.” They also asked us to consider deleting the quotes about how providers use the law to overcome school administrator’s refusal to enroll YWLS.

In the revised manuscript, we have edited this section of the results to provide additional quotes from providers on the ways in which they perceive schools to be hostile places for YWLS. The additional data appears in on pages 12-13, which is then followed by the two quotes about how providers rely on public laws and legal actors to enroll YWLS in schools.

22. The Editor asked us to clarify the terms courtesy stigma, institutional stigma, cultural racism and intersectional stigma. In so doing, the Editor also asked us to reserve value judgements for discussion where we can define these terms, or to define our use of the terms earlier in the text, e.g., in the introduction.

We included the definition of intersectional stigma on page 4 of the original manuscript in the following sentence: “Recent scholarship has focused on intersectional stigma, i.e., the presence of multiple devalued social markers, such as race, class, and sexual identity, that interact to produce layered experiences of stigma and discrimination [19–22].” Courtesy stigma was likewise defined on page 4 of the manuscript in the following sentence: “Because stigma is socially produced, public stigma can be transferred to people affiliated with stigmatized groups (i.e., courtesy stigma), including health and social service providers [32–34].” We also have elaborated on our original description of cultural racism in the manuscript. In the revised introduction, we now define antiziganism and Romaphobia, which has expanded the discussion of cultural racism (see page 6 of the revised manuscript). Finally, institutional and structural stigma are now defined on page 5.

23. The Editor was concerned that we provided too much interpretation in the results section and asked us to delete the following sentence from the results: “In their responses, providers endorsed patriarchal gender norms by framing ethnic minority YWLS and their families as deviating from traditional family structures.” We have deleted this sentence. This interpretation also was deleted from the discussion section in light of other changes made to the revised manuscript.
24. The Editor was concerned that we were making leaps from participants narrating their lived experiences to calling such experiences racist. The Editor asked us to label quotes in the results as negative stereotypes and to consider the extent to which there may be kernels of truth in provider’s statements that ethnic minority girls experience child marriages or begin their sex lives early.

In the prior version, we interpreted these statements as forms of cultural racism because these stereotypes have been documented in numerous studies. We also are sensitive to the Editor’s point about labeling providers racist, which was not our intention. In the revised results, we use the language of negative stereotypes in the results and discussion. In the discussion, we have further contextualized providers’ stereotypes about ethnic minority youth in the broader literature. We also note that for some providers, there was a tendency to over-emphasize the role of families or cultures rather than the larger social contexts that are correlated with early marriage, early sex, and commercial sex—a phenomenon documented by numerous other scholars (see page 24).

25. The Editor asked us to edit the following sentence: “For many youth, self-stigma manifested as feelings of shame, negative self-worth, and a heightened sense being different from other youth, e.g., “They are ashamed because they think they are behind, as they never went to school. They are different than other children and it affects everything” (Manager, Respondent 13). to: “For many youth, self-stigma was said to manifest as feelings of shame, negative self-worth….” This change has been made (see page 20).
26. Related to the above point, the Editor requested that we edit the manuscript to clarify that we are discussing the opinions of the key informants and not of the YWLS. We have made this edit throughout the revised manuscript.
27. The Editor asked us to use lowercase letters for job positions of respondents. We have made this change throughout the revised manuscript.
28. The Editor posed several questions about the types of YWLS in Georgia, including: “Are there children on the street that are not ethnic minority and is there any inclination that their experience is different from those that are ethnic minorities? Why have the authors not assessed issues around all YWLS but focused on ethnic minority?”

In the original manuscript, we had described the different types of ethnic groups of youth who live and/or work on the street, including native Georgian, Russian, European, Azeri-Kurdish, and two distinct Romani groups of youth (pages 2-4). We discuss stigma towards ethnic minority YWLS in our findings because this emerged as a strong theme in the data. The interview questions did not ask providers about the differences between different groups of youth. Through our analysis, however, we identified that providers perceived that while all youth were subjected to public stigma, some reported that stigma and discrimination were worse for ethnic minority youth. The focus of this paper was thus driven by the data and the findings reflects the major themes that we identified in our thematic analysis.

**Discussion**

29. Referring to the following sentence in the discussion, “The processes described by providers follow those originally outlined by Goffman [15].” The Editor stated that was better to give the outline of the process than allude to it. We outlined the processes in the introduction so did not repeat them here. In response, we have edited this sentence to include the labeling, stereotyping, separation and discrimination processes outlined by Goffman (see page 22).
30. In response to the following sentence, “We also find support for the presence of intersectional [21], institutional, and structural stigma [43].” the Editor stated: “As already mentioned, authors have not defined any of these terms in the manuscript.” Please see our response to item 22 above.
31. In response to this statement: “Importantly, providers did not report elevated stigma towards YWLS who are from Russia or other European countries, who also are part of the population of YWLS in Georgia [4,65],” the Editor noted that: “It will be interesting to know from literature if persons of Romani or Azeri-Kurdish origin who are not living on the street experience any form of stigmatization/discrimination by the virtue of their places of origin.”

In the prior submission, we included a discussion of discrimination towards Romani in other countries. We have elaborated on this phenomenon to page 6 of the revised manuscript. In the revised manuscript, we now include this sentence at the end of the results section on page 16. In the discussion section, we now add a brief sentence on the need for future research on stigma towards Azeri-Kurds (see page 23).

32. In response to the following statement, “Although providers clearly recognized stigma as a negative animating force in the lives of YWLS, especially those from ethnic minority groups, a subset of providers also endorsed stigmatizing beliefs towards ethnic minority youth,” the Editor stated: “Again, the argument of a subset of providers need to be made strong. Subset implies that there is some common underlying characteristics, So, one might expect age, profession, ethnicity etc. If there is no commonalty among those with the said opinion, then they are not really subset in the context of this research. In that case the authors would need to reword their statements to read more appropriately like: “…some of the providers seemed to endorse stigmatizing beliefs…”

We have removed the word subset and now state “some of the providers seemed to express negative beliefs…”

33. In response to the sentence that: “We observed the same discourse among providers in their descriptions of Roma children being exposed to dangerous family environments,” the Editor asked us to consider way that living on the street, irrespective of ethnicity, exposes youth to dangerous living experiences that may include a dangerous family environment. In addition, the Editor suggested we consider adding a conceptual framework that shows the dynamics of stigma for YWLS.

We agree with the Reviewer that living on the street or being in a dangerous family environment can expose youth to a host of negative living experiences, irrespective of their ethnicity; this is now noted on page 24. While we appreciate the Reviewer’s encouragement to develop a conceptual framework that shows the dynamics of stigma, we do not believe that we have sufficient data to develop such a framework. To develop a robust enough framework, we believe that we would need data from youth and from a larger sample of providers. Nevertheless, we are sensitive to the Editor’s comments. In the revised manuscript, we have expanded our discussion of the discursive framing of risk around ethnic minority YWLS (see page 24). In addition, we have edited the discussion to ensure that we are not drawing any causal inferences. Finally, we have expanded the recommendations on future research section to include longitudinal research than can help to elucidate the causal mechanisms between stigma and biopsychosocial health outcomes of YWLS over time (see page 27).

34. The Editor stated that we did not provide evidence in the results to support the following statement: “Notably, provider stigma towards ethnic minority YWLS was observed among a number of staff with advanced educational degrees, e.g., social workers and psychologists, as well as peer educators—young people with their own history of working and/or living on the street.”

In the results, we included the employment position for each quote, e.g., social worker, psychologist, outreach worker. In so doing, we are able to state that some of the social workers and psychologists, e.g., key informants with advanced degrees, and peer outreach workers, e.g., key informants with their own history of living and/or working on the street, did indeed express negative stereotypes, a type of stigma, towards YWLS. We disagree with the Editor that this information is not in the results.

35. The Editor felt that some of the claims in the discussion extended beyond the scope of the data and highlighted this sentence as an example: “It is possible that the negative views displayed here shape who providers engage in services, highlighting another pathway through which stigma may shape the social exclusion of ethnic minority YWLS.”

We were not attempting to claim that we observed this relationship in our data; rather, we were stating that this could be a potential pathway, which has been observed in prior work. In the revised manuscript, we have edited this sentence to be clear that we are not generalizing beyond the scope of the data (see page 25).

36. In response to the following statement, “Our study adds to the growing body of literature on the role of courtesy stigma [32–34],” the Editor wrote: “Again, key terms are not defined anywhere in the manuscript.” Please see our response to Item 22 above.
37. The Editor stated: “As a general comment, a major limitation in this study is that there was no focus on YWLS of non-minority ethnicity. So, it cannot be inferred from this study if the experience of minority YWLS described is unique, or other categories of YWLS also experience similar stigma.”

We agree that a limitation of the study was that we did not include youth. We had previously mentioned this in the limitations section. To address the Editor’s concern, we have noted in the revised manuscript that future research with Georgian and ethnic minority YWLS is needed to better understand how stigma is experienced among each group (see page 26).

38. During the revision, we also reviewed for typographical and grammatical errors. We also would like to shorten the title of the manuscript to, “They walk with bare feet on broken glass,” as we believe the shorter title is more appropriate than the longer quote we originally had.

We believe that the revisions have strengthened the manuscript and hope that the revised is now acceptable for publication. Thank you for your careful review.

Sincerely,

Shorena Sadzaglishvili, PhD, MSW

Professor

Ilia State University

ISU Room E327

Cholokashvili 3/5 Avenue

Tbilisi 0162, Georgia
