# Supplementary material for: “How can you kiss and touch this child and show affection towards her? What kind of woman are you?”: Provider perspectives on stigma towards native and ethnic minority street-connected youth in the Republic of Georgia
Source: PLoS One. 2023 Jun 2;18(6):e0286710. doi: 10.1371/journal.pone.0286710 (PMC10237414; doi:10.1371/journal.pone.0286710)
Supplement: Attachment — Submitted filename: Response to Reviewers FINAL PLOS One.docx [file pone.0286710.s003.docx]

April 21, 2023

PONE-D-22-13919R2

Anastassia Zabrodskaja, Ph.D.
Academic Editor
PLOS ONE

Dear Dr. Zabrodskaja,

Enclosed, please find the revision to Manuscript PONE-D-22-13919R2. We appreciate the Reviewers’ additional comments, and have attempted to respond to all suggestions. In most cases, we made the suggested changes. In one instance, a requested revision contradicted changes requested by the prior Academic Editor, who had provided a lengthy list of requested revisions. Nevertheless, we have made the requested change here, as we believe that the Reviewer’s suggestions improved the clarify of the manuscript. In total, there was only one suggestion that we were not able to pursue, and we note this below. Finally, as required, we have made all edits in Track Changes. The data remain available in the repository and we hope the changes address the different conclusions of the Reviewers about the conclusions and findings.

**Responses to Reviewers**

Reviewer #3: We appreciate that Reviewer #3 determined that the paper addresses an understudied topic in the extant global health literature. We thank the Reviewer for the additional comments and opportunity to strengthen the manuscript.

1. Reviewer 3 did not believe that the current quote, e.g., “they walk with bare feet on broken glass” shed light on the main themes of the paper and suggested we select another quote to use in the title.

Thank you for this suggestion. We reviewed the manuscript and have selected a new quote for the title: *“How can you kiss and touch this child and show affection towards her? What kind of woman are you*?” We selected this quote because we believe that it reflects public stigma directed towards youth and courtesy stigma that providers report experiencing in their work with young people who live and/or work on the street.

2. The author requested that we better specify the term “social contexts” in the following phrase: “provider perspectives on the social contexts surrounding the delivery of services.”

Thank you for this comment. On page 8 of the revised manuscript, we have added text to clarify this term. The sentence now reads: “The purpose of the interviews was to understand provider perspectives on the social networks and HIV prevention needs of YWLS, their experiences delivering services to YWLS, and their recommendations for developing trusting relationships with YWLS.” We also have clarified this aspect of the interview guide on page 10.

3. The abstract uses the term 'courtesy stigma' which won't be clear to many readers unless you add an example or definition. We have added a definition of courtesy stigma to the abstract.

Reviewer #4: We appreciate that Reviewer #4 deemed that we were responsive to the prior Editor’s comments and that the revisions had strengthened the rigor of the results and conclusions. We also welcome the Reviewer’s additional suggestions to strengthen the methodology section.

1. Reviewer 4’s primary concern was with the methodology section. They stated that the “opening sentence doesn’t fit well with the first paragraph. The opening paragraph deals with the sampling plan, which ought not to be. The normal is to start with the research design, followed by the population of the study, the sampling plan, the method of data collection, the instruments used and then how the data were analysed.”

To address this concern, we have moved the paragraph titled “Present study” in the previous revision to the start of the methods. We also have edited this paragraph to address the Reviewer’s concern. As the Reviewer notes, this was a qualitative exploratory study. We now state this and have added the following paragraph to the start of the methods on pages 8-9.

“This qualitative exploratory study was conducted during the formative phase of See the Future in Us, a multi-method study examining the social networks and HIV prevention needs of YWLS [10]. During this exploratory phase, we conducted individual in-depth interviews with key informants employed in social service organizations working with YWLS in Tbilisi and Rustavi, Georgia. The purpose of the interviews was to understand provider perspectives on the HIV prevention needs of YWLS, their experiences delivering services to YWLS, and their recommendations for developing trusting relationships with YWLS. Key informants included social workers, psychologists, logistics officers who supported mobile outreach teams, managers of social service organizations, and peer educators who were young adults with a prior history of living and/or working on the street.”

2. The Reviewer asked us to better specify the information on the population from which the sample of 22 service providers was drawn, including the technique adopted in sample selection, whether the 22 participants were selected from a known population (such as the total number of social service organisations), and the selection method (e.g., randomisation, purposive, or convenience). If the population is not known, the Reviewer asked us to specify how the organisations were selected and how the participants identified, e.g., snowballing or through a documented list of service providers.

Thank you for the opportunity to clarify the sampling approach. We developed a sampling frame based on the 10 known governmental and non-governmental organizations that provide services to YWLS in Tbilisi and Rustavi. From this list, we used a purposive sampling method to identify and recruit potentially eligible key informants (KIs) who worked with YWLS in each agency. On page 9, we have revised the text to state:

“We developed a sampling frame based on the 10 known governmental and non-governmental organizations that provide services to YWLS in Tbilisi and Rustavi. From this list, we used a purposive sampling method to identify and recruit potentially eligible key informants (KIs) who worked with YWLS in each agency. KIs were eligible to participate if they (1) were 18 years or older, (2) worked in an agency providing services to YWLS; and (3) had direct contact with YWLS through their work. All eligible staff in each agency were contacted via telephone and invited to participate in an individual interview. During recruitment, the local research team explained the purpose of the study and all participants provided written informed consent. In total, 95% of all identified and eligible staff were recruited into the study. None of the study participants had an official relationship with the study and were not involved in developing the study or interpreting its results.”

3. Reviewer 4 stated: “The choice of in-depth interviews with an interview guide comprising structured and unstructured questions is apt. The same goes for the choice of thematic analysis for data analysis. What is missing is the way these are presented in the paper. I, therefore, suggest that this section is reworked, adopting the recommended systematic approach. I believe that such an approach will make comprehension easier.”

The presentation of the results have undergone considerable revisions during the review process. In our original submission, we had listed the themes at the start of the results. In the last revision, the prior Academic Editor requested we remove this text and just present the results. We are not aware of a recommended systematic approach to presenting the results and could not find one on the PLOS One webpages. However, we agree that the presentation of the results could be streamlined to improve reader comprehension. For this revision, the first and last authors reviewed prior articles published by PLOS One that included thematic analysis of qualitative data. Through reading these articles, we did not observe a single approach to presenting results. However, we noticed that most articles state the number of themes and subthemes at the start of the results, and list the primary themes—thereby providing readers with an early description of the results. In our revision, we have adopted this approach. To further strengthen the results, we revisited the results of the qualitative data analysis to enrich the titles for each theme, further grounding each theme in participant’s words. As we made these revisions, we also noticed that our subthemes were not clearly labeled; rather, they were included as part of a major theme. To further improve comprehension and clarity, we now clearly identify the subthemes that were present in relevant identified themes. On page 12, the revised introduction to the results now states:

“We identified five themes and five subthemes. The primary themes are: (1) Already labeled objects who spoil everything: Public and institutional stigma directed towards YWLS; (2) Dirty and despicable criminals: Intersectional stigma for ethnic minority YWLS; (3) Provider stereotypes about ethnic minority youth: Labeling, stereotyping and separating “us” from “them;” (4) Youth fear, anxiety, and distrust: Provider perspectives on how public stigma affects YWLS; and (5) Difficult working environments: Provider encounters with courtesy stigma. Each theme and any relevant subthemes are discussed below, with illustrative quotes from interviews along with the professional role of each respondent.”

4. The final concern raised by Reviewer #4 regarded our previous claim of “high” levels of stigma in the section on “Difficult working environments: provider encounters with courtesy Stigma”. The Reviewer stated, “Though this statement has been amended (deleted), it is truly difficult to showcase the level of significance in a qualitative study. However, there is a way out. You may use word cloud, which will clearly indicate the most prominent theme of keyword in your thematic analysis. This is in addition to using enough quotes from the participants. If you still wish to retain that statement (which, in my opinion, contributes significantly to the result, I suggest you present it in a word cloud.”

We agree with the Reviewer that it is difficult to showcase the level of significance in a qualitative study. While we appreciate the suggestion to utilize a word cloud, we are not sure how to do this given that we coded the data in Georgian and would need to translate any word cloud. In reviewing the manuscript, we are comfortable with retaining the prior revision to not make this claim about high levels of stigma. We also have changed high to strong in other parts of the manuscript.

We believe that the revisions have once again strengthened the manuscript and hope that the third revision is now acceptable for publication. Thank you, again, for the thoughtful reviews.

Sincerely,

Shorena Sadzaglishvili, PhD, MSW

Professor

Ilia State University

ISU Room E327

Cholokashvili 3/5 Avenue

Tbilisi 0162, Georgia
