# Supplementary material for: “How can you kiss and touch this child and show affection towards her? What kind of woman are you?”: Provider perspectives on stigma towards native and ethnic minority street-connected youth in the Republic of Georgia
Source: PLoS One. 2023 Jun 2;18(6):e0286710. doi: 10.1371/journal.pone.0286710 (PMC10237414; doi:10.1371/journal.pone.0286710)
Supplement: Peer review history

# Peer review: Decision Letter 0

**DOI:** 10.1371/journal.pone.0286710.r001

**Authors:** Thomas E. Guadamuz

18 Aug 2022

PONE-D-22-13919“They walk with bare feet on broken glass, they are not developing normally, and they are not learning anything”: Provider perspectives on stigma towards native and ethnic minority street-connected youth in the Republic of GeorgiaPLOS ONE

Dear Dr. Sadzaglishvili,

Thank you for submitting your manuscript to PLOS ONE. After careful consideration, we feel that it has merit but does not fully meet PLOS ONE’s publication criteria as it currently stands. Therefore, we invite you to submit a revised version of the manuscript that addresses the points raised during the review process.

Please submit your revised manuscript by Oct 02 2022 11:59PM. If you will need more time than this to complete your revisions, please reply to this message or contact the journal office at plosone@plos.org. When you're ready to submit your revision, log on to https://www.editorialmanager.com/pone/ and select the 'Submissions Needing Revision' folder to locate your manuscript file.

Please include the following items when submitting your revised manuscript:

- A rebuttal letter that responds to each point raised by the academic editor and reviewer(s). You should upload this letter as a separate file labeled 'Response to Reviewers'.
- A marked-up copy of your manuscript that highlights changes made to the original version. You should upload this as a separate file labeled 'Revised Manuscript with Track Changes'.
- An unmarked version of your revised paper without tracked changes. You should upload this as a separate file labeled 'Manuscript'.

If you would like to make changes to your financial disclosure, please include your updated statement in your cover letter. Guidelines for resubmitting your figure files are available below the reviewer comments at the end of this letter.

If applicable, we recommend that you deposit your laboratory protocols in protocols.io to enhance the reproducibility of your results. Protocols.io assigns your protocol its own identifier (DOI) so that it can be cited independently in the future. For instructions see: https://journals.plos.org/plosone/s/submission-guidelines#loc-laboratory-protocols. Additionally, PLOS ONE offers an option for publishing peer-reviewed Lab Protocol articles, which describe protocols hosted on protocols.io. Read more information on sharing protocols at https://plos.org/protocols?utm_medium=editorial-email&utm_source=authorletters&utm_campaign=protocols.

We look forward to receiving your revised manuscript.

Kind regards,

Thomas E. Guadamuz, Ph.D., M.H.S.

Academic Editor

PLOS ONE

Journal Requirements:

When submitting your revision, we need you to address these additional requirements.

1. Please ensure that your manuscript meets PLOS ONE's style requirements, including those for file naming. The PLOS ONE style templates can be found at

https://journals.plos.org/plosone/s/file?id=wjVg/PLOSOne_formatting_sample_main_body.pdf  and

https://journals.plos.org/plosone/s/file?id=ba62/PLOSOne_formatting_sample_title_authors_affiliations.pdf

2. In your Data Availability statement, you have not specified where the minimal data set underlying the results described in your manuscript can be found. PLOS defines a study's minimal data set as the underlying data used to reach the conclusions drawn in the manuscript and any additional data required to replicate the reported study findings in their entirety. All PLOS journals require that the minimal data set be made fully available. For more information about our data policy, please see http://journals.plos.org/plosone/s/data-availability.

Upon re-submitting your revised manuscript, please upload your study’s minimal underlying data set as either Supporting Information files or to a stable, public repository and include the relevant URLs, DOIs, or accession numbers within your revised cover letter. For a list of acceptable repositories, please see http://journals.plos.org/plosone/s/data-availability#loc-recommended-repositories. Any potentially identifying patient information must be fully anonymized.

Important: If there are ethical or legal restrictions to sharing your data publicly, please explain these restrictions in detail. Please see our guidelines for more information on what we consider unacceptable restrictions to publicly sharing data: http://journals.plos.org/plosone/s/data-availability#loc-unacceptable-data-access-restrictions. Note that it is not acceptable for the authors to be the sole named individuals responsible for ensuring data access.

We will update your Data Availability statement to reflect the information you provide in your cover letter.

3. We note that you have stated that you will provide repository information for your data at acceptance. Should your manuscript be accepted for publication, we will hold it until you provide the relevant accession numbers or DOIs necessary to access your data. If you wish to make changes to your Data Availability statement, please describe these changes in your cover letter and we will update your Data Availability statement to reflect the information you provide.

4. Please include your full ethics statement in the ‘Methods’ section of your manuscript file. In your statement, please include the full name of the IRB or ethics committee who approved or waived your study, as well as whether or not you obtained informed written or verbal consent. If consent was waived for your study, please include this information in your statement as well.

5. Thank you for stating the following in the Acknowledgments Section of your manuscript:

“This research was funded by the Shota Rustaveli National Science Foundation. (SRNSFG) [#FR 17_31]”

We note that you have provided additional information within the Acknowledgements Section that is not currently declared in your Funding Statement. Please note that funding information should not appear in the Acknowledgments section or other areas of your manuscript. We will only publish funding information present in the Funding Statement section of the online submission form.

Please remove any funding-related text from the manuscript and let us know how you would like to update your Funding Statement. Currently, your Funding Statement reads as follows:

“This research was funded by the Shota Rustaveli National Science Foundation. (SRNSFG) [#FR 17_31]

Principal Investigator: Shorena Sadzaglishvili”

Please include your amended statements within your cover letter; we will change the online submission form on your behalf.

6. Thank you for stating the following financial disclosure:

“This research was funded by the Shota Rustaveli National Science Foundation. (SRNSFG) [#FR 17_31]

Principal Investigator: Shorena Sadzaglishvili”

Please state what role the funders took in the study.  If the funders had no role, please state: "The funders had no role in study design, data collection and analysis, decision to publish, or preparation of the manuscript."

If this statement is not correct you must amend it as needed.

Please include this amended Role of Funder statement in your cover letter; we will change the online submission form on your behalf.

7. Thank you for stating the following in your Competing Interests section: 

“No authors have competing interests”

Please complete your Competing Interests on the online submission form to state any Competing Interests. If you have no competing interests, please state "The authors have declared that no competing interests exist.", as detailed online in our guide for authors at http://journals.plos.org/plosone/s/submit-now

 This information should be included in your cover letter; we will change the online submission form on your behalf.

8. Please review your reference list to ensure that it is complete and correct. If you have cited papers that have been retracted, please include the rationale for doing so in the manuscript text, or remove these references and replace them with relevant current references. Any changes to the reference list should be mentioned in the rebuttal letter that accompanies your revised manuscript. If you need to cite a retracted article, indicate the article’s retracted status in the References list and also include a citation and full reference for the retraction notice.

Additional Editor Comments :

Dear Dr. Sadzaglishvili,

Firstly, apologies for the delay in the review process. It was difficult getting qualified reviewers, especially during these summer months. I have now received comments from both reviewers and they believe that your manuscript is interesting and will contribute significantly to the literature. Both reviewers have relevant and constructive suggestions that you may find useful. Please go through each comment and provide a response or rebuttal.

Regards,

Thomas

[Note: HTML markup is below. Please do not edit.]

Reviewers' comments:

Reviewer's Responses to Questions

**Comments to the Author**

1. Is the manuscript technically sound, and do the data support the conclusions?

The manuscript must describe a technically sound piece of scientific research with data that supports the conclusions. Experiments must have been conducted rigorously, with appropriate controls, replication, and sample sizes. The conclusions must be drawn appropriately based on the data presented. 

Reviewer #1: Yes

Reviewer #2: Yes

**********

2. Has the statistical analysis been performed appropriately and rigorously? 

Reviewer #1: N/A

Reviewer #2: N/A

**********

3. Have the authors made all data underlying the findings in their manuscript fully available?

The PLOS Data policy requires authors to make all data underlying the findings described in their manuscript fully available without restriction, with rare exception (please refer to the Data Availability Statement in the manuscript PDF file). The data should be provided as part of the manuscript or its supporting information, or deposited to a public repository. For example, in addition to summary statistics, the data points behind means, medians and variance measures should be available. If there are restrictions on publicly sharing data—e.g. participant privacy or use of data from a third party—those must be specified.

Reviewer #1: No

Reviewer #2: No

**********

4. Is the manuscript presented in an intelligible fashion and written in standard English?

PLOS ONE does not copyedit accepted manuscripts, so the language in submitted articles must be clear, correct, and unambiguous. Any typographical or grammatical errors should be corrected at revision, so please note any specific errors here.

Reviewer #1: Yes

Reviewer #2: Yes

**********

5. Review Comments to the Author

Please use the space provided to explain your answers to the questions above. You may also include additional comments for the author, including concerns about dual publication, research ethics, or publication ethics. (Please upload your review as an attachment if it exceeds 20,000 characters)

Reviewer #1: 1. the paper aims to reveal provider perspectives on stigma toward YWLS but the finding of this part is too thin when compared with the content on stigma from society towards YRLS. Above all, it's not clear enough to judge what the KIs said about YWLS are stigma perception or knowledge from frontline experiences working on the street. There is little evidence to support the claim on par 2 page 10 which concludes that "we found that providers themselves report high levels of stigma toward ethnic minority YWLSW",

2. the term semi-structured in-depth interview should be reconsidered. the problem is if the interview question is structured, how could it be for an in-depth probe? If a semistructured form was used, then quantitative data would be expected. In addition, how the 22 informants were included should be elaborated- i.e., officially or unofficially, accidental or through network relationship?

3. positionality issue: interesting that all researchers are adult, and social service providers, and none has personal histories living or working on the street, how would these backgrounds play roles in your data analysis and interpretation, what are obstacles, difficulties or lessons learned moving forth and back between insiders and outsider? Adding this in the text will be useful.

4. Par2, page 21, states that "a subset of providers also endorsed stigmatizing beliefs towards ethnic minority youth." It will be useful to discuss who are they and what factors account for their beliefs. On the other hand, for those who do not have such beliefs, who are they and what makes them different.

5. In conclusion, this paper is good in filling the gap of knowledge on this issue but still does not fully answer what intended to. Good in this paper is the literature review part. Yet it will be much better if more relevant studies on provider perspectives on stigma towards YWLS are added.

Reviewer #2: “They walk with bare feet on broken glass, they are not developing normally, and they are not learning anything”: Provider perspectives on stigma towards native and ethnic minority street- connected youth in the Republic of Georgia

This was an excellent paper and a pleasure to read. Thank you for the opportunity to review it. The authors do a great job of locating the study within the literature around stigma. The methods are appropriate, well described and sensible for answering the research question. The results are robust and illuminate the key ways stigma impacts street-connected youth in the Republic of Georgia. The discussion appropriately summarises the evidence and locates it within the broader literature. I have only minor comments which I hope will strengthen the publication.

Introduction

1. Spaces need to be added between words and references in a few areas, the below sentence is illustrative:

"...including violence and victimization[5], economic and sexual exploitation[6], poor mental health[7], substance abuse[8], and sexually transmitted infections, including HIV/AIDS[9]."

2. A word appears to be missing at the end of the below sentence (maybe ‘life’?)

"Currently, social service delivery for YWLS in Georgia focuses on both preventing youth from entering street life and on reintegrating young people into mainstream social [4]."

3. Overall the introduction is pretty long and could be made a bit more concise. This will help make the paper engaging to the widest possible audience.

Methods

4. More information could be provided about how the topic guide was developed. E.g. was this from other similar literature? Or community engagement?

Results

5. Throughout the results section there are many references to ‘family’. It seems implicit in this paper that some of the stigma towards street connected youth emerges from traditional conceptions of family drawn from patriarchal norms. This comes up in several of the subheadings and could be made clearer as a thread that connects multiple forms of stigma for this population. This could then be tied together in the discussion

Discussion

6. See above point re: family and patriarchal norms.

7. The discussion left me wondering if the authors have thoughts on what types of interventions or approaches could be taken to reduce stigma. This isn’t essential but it is worth considering adding a short paragraph on this.

**********

6. PLOS authors have the option to publish the peer review history of their article (what does this mean?). If published, this will include your full peer review and any attached files.

If you choose “no”, your identity will remain anonymous but your review may still be made public.

**Do you want your identity to be public for this peer review?** For information about this choice, including consent withdrawal, please see our Privacy Policy.

Reviewer #1: No

Reviewer #2: No

**********

[NOTE: If reviewer comments were submitted as an attachment file, they will be attached to this email and accessible via the submission site. Please log into your account, locate the manuscript record, and check for the action link "View Attachments". If this link does not appear, there are no attachment files.]

While revising your submission, please upload your figure files to the Preflight Analysis and Conversion Engine (PACE) digital diagnostic tool, https://pacev2.apexcovantage.com/. PACE helps ensure that figures meet PLOS requirements. To use PACE, you must first register as a user. Registration is free. Then, login and navigate to the UPLOAD tab, where you will find detailed instructions on how to use the tool. If you encounter any issues or have any questions when using PACE, please email PLOS at figures@plos.org. Please note that Supporting Information files do not need this step.

# Author comment: Author response to Decision Letter 0

**DOI:** 10.1371/journal.pone.0286710.r002

6 Oct 2022

September 24, 2022

Thomas E. Guadamuz, Ph.D., M.H.S.

Academic Editor, PLOS ONE

PONE-D-22-13919

“They walk with bare feet on broken glass, they are not developing normally, and they are not learning anything”: Provider perspectives on stigma towards native and ethnic minority street-connected youth in the Republic of Georgia

Dear Dr. Guadamuz,

We have appended our revision for PONE-D-22-13919. We appreciate the comments from the Reviewers and the opportunity to revise the manuscript. Below, please find our responses to journal-specific requests, as well as our specific comments and rebuttals to Reviewer comments. As requested, we have appended a marked up copy titled 'Revised Manuscript with Track Changes' and an unmarked version titled 'Manuscript'.

Below are our responses to journal requirements. 

1. Please ensure that your manuscript meets PLOS ONE's style requirements, including those for file naming. 

The manuscript has been revised to meet PLOS ONE’s style requirements. 

2. In your Data Availability statement, you have not specified where the minimal data set underlying the results described in your manuscript can be found. 

You can find minimal data set on the Ilia State University repository. 

http://eprints.iliauni.edu.ge/cgi/users/home?screen=EPrint%3A%3AView&eprintid=11133

3. As noted above, we uploaded a minimal data set to: 

http://eprints.iliauni.edu.ge/cgi/users/home?screen=EPrint%3A%3AView&eprintid=11133

4. Please include your full ethics statement in the ‘Methods’ section of your manuscript file. 

We have added the full name of the IRB ethics committee who approved the study to page 8. It now states: “All study protocols were approved by the Ilia State University Ethics Committee on June 26, 2018 (R/333-18).” In the revised submission, the statement regarding written informed consent is on page 8. 

5. Thank you for stating the following in the Acknowledgments Section of your manuscript: “This research was funded by the Shota Rustaveli National Science Foundation. (SRNSFG) [#FR 17_31]”. We note that you have provided additional information within the Acknowledgements Section that is not currently declared in your Funding Statement. Please note that funding information should not appear in the Acknowledgments section or other areas of your manuscript. We will only publish funding information present in the Funding Statement section of the online submission form. Please remove any funding-related text from the manuscript and let us know how you would like to update your Funding Statement.

We removed funding information text from the manuscript. However, we need to include this statement in the Funding Statement Section: 

“This research was funded by the Shota Rustaveli National Science Foundation. (SRNSFG) [#FR 17_31]

Principal Investigator: Shorena Sadzaglishvili”

Please change the online submission form according to this information. 

6. As requested, we now clarify the role of the funding agency in the study. The amended Role of Funder statement is below: 

“The funders had no role in study design, data collection and analysis, decision to publish, or preparation of the manuscript.”

7. As requested, we clarify the Competing Interests below and have updated the online submission form:

“The authors have declared that no competing interests exist.”

8. We reviewed the reference list, as requested. No references were deleted and no references are based on retracted papers. We did, however, add references in response to requests from Reviewer 1. Specifically, references 76 to 87 are new.

Reviewer Comments 

Reviewer 1 provider several suggestions to strengthen the literature, methods, and analytic interpretations. Below, we summarize and respond to Reviewer One’s specific comments.

1. Reviewer 1 believed that our data on provider stigma towards ethnic minority YWLS was too thin and that it wasn’t clear enough to judge that what KIs said about YWLS was stigma perception or knowledge from frontline experiences working on the street. The Reviewer concluded that there was little evidence to support the claim on par 2 page 10 that “we found that providers themselves report high levels of stigma toward ethnic minority YWLSW.”

We appreciate the opportunity to clarify the nature and strength of the findings that a subset of providers demonstrated stigma towards ethnic minority YWLS, as opposed to repeating stigmatizing attitudes and beliefs held by others. On page 10 of the revised manuscript, we have edited the introduction to the results to state:

“First, we found that providers described high levels of public stigma directed towards YWLS, with public stigma manifesting as institutional stigma to limit youth access to key services like schools and medical care. Second, we found that cultural racism [57] produces intersectional stigma [21,47] for Roma and Azeri-Kurdish youth, who encounter stigma based on their status as street-children and as ethnic minorities. In addition, we found that a subset of providers who discussed public stigma towards YWLS also reported negative attitudes and beliefs about ethnic minority YWLS, illustrating how cultural racism may manifest as provider stigma for multiply marginalized YWLS. Providers described how high levels of public stigma negatively impact YWLS, who often fear and distrust large segments of Georgian society. Finally, we found that providers who serve YWLS describe encountering courtesy stigma, which creates a stressful service delivery context. Each theme is discussed below, with illustrative quotes from interviews along with the professional role of each respondent.”

To further clarify how the data supports our claim, we have reorganized the results section that describes provider stigma towards ethnic-minority youth (see pages 14-17). In the revised results, we now lead with the data describing how providers contrasted Georgian youth with ethnic minority youth, noting how the comparison reveals a difference in attitudes and beliefs between the two groups of YWLS. In addition, we have more clearly noted that these beliefs were expressed among a subset of providers and not all providers. We believe that these changes address the Reviewer’s concerns and allow us to clarify the findings.

2. The Reviewer posed several methodological questions. First, they asked us to reconsider using the term semi-structured in-depth interview; here, the Reviewer thought that one could not use in-depth probes if the interview question was structured. They also noted quantitative data would be expected if a semi-structured form was used. In addition, the Reviewer wanted additional information on how the 22 key informants were included in the sample, i.e., where the recruited officially or unofficially, accidental, or through network relationships?

We used a semi-structured interview guide for the key informant interviews. The questions were developed by the Research Team based on the extant literature and stakeholder feedback and designed to elicit provider perspectives on: (1) the social network characteristics of YWLS, (2) youth’s involvement in substance use and sexual behaviors that may heighten the likelihood for HIV transmission, and (3) the social contexts of youth engagement and service delivery. During the interviewers, data collectors posed questions to each key informant and used probes to elicit additional details as needed. Semi-structured interview guides are ideal for qualitative research as they provide structure and flexibility. As opposed to a structured guide, where questions may be close-ended, questions in a semi-structured are typically open-ended. The open-ended nature of the questions allowed us to elicit thicker and more nuanced descriptions from key informants and to then analyze these data for emergent themes across interviews. On page 8 of the revised manuscript, we note that we used open-ended questions and that interviewers used probes to elicit additional details from providers.

Although quantitative approaches can be used to analyze qualitative data, this was not the purpose of the present study. Rather, we sought to qualitatively explore how providers describe their work with street connected youth. As such, we did not apply a quantitative approach to analyzing the data. 

Finally, the informants were recruited from all existing social service organizations in two major cities, Tbilisi and Rustavi. We do not provide names of the organizations so as to ensure participant confidentiality. The key informants did not have an official relationship with the study and were not involved in developing the study or interpreting study results. We have added this information to page 8.

3. Reviewer 1 asked us to consider how including youth or adults with experiences of living and/or working on the street would have shaped our analysis, interpretation and any lessons learned. 

We appreciate the Reviewer’s point and agree that including YWLS would have yielded additional insights on our role as outsiders. In the limitations section, we acknowledge this point and discuss the importance of involving youth with lived experience as members of the research team. In so doing, we note the need for youth participatory action research with YWLS in Georgia, which would include youth as full partners in the research endeavor. Here, we briefly note that prior research using YPAR with YWLS has yielded important benefits and is important for giving youth a voice in the practices, programs and policies that shape their lives (please see page 25). 

4. Reviewer 1 noted that it would be useful to discuss who the providers were that expressed stigmatizing beliefs towards YWLS and to describe the factors that account for their beliefs, as well as what differentiates them from providers who did not express these beliefs. 

In the original manuscript, we included the professional role of each provider, e.g., social worker, psychologist, coordinator, peer educator, etc. We did not provide additional details on the key informants, as this information would risk deductive disclosure and a potential loss of confidentiality for those providers who agreed to participate. In addition, because the interview guide was not designed to focus on stigma, we did not have follow up questions that elucidate how providers came to hold these beliefs and the factors that might differentiate between those who did and did not endorse stigmatizing beliefs towards YWLS. As such, we are not able to identify what factors account for the different levels of stigmatizing beliefs among providers. Nevertheless, we believe this work is necessary to move forward with programs to address provider stigma towards different groups YWLS. In the discussion section, we note the need for future research in this area (see page 23).

5. The Reviewer noted that while the paper addressed the knowledge gap on provider perspectives on stigma directed towards YWLS, it needed additional revision to fulfill the stated purpose. While they stated that the literature review was good, they requested that we add more relevant studies on provider perspectives on stigma towards YWLS.

To date, few studies have examined the perspectives of Georgian providers on stigma towards YWLS, a gap this study seeks to address. Reviewer 2 also asked us to condense the literature review section. Nevertheless, we added information on provider stigma towards YWLS, reporting details of a 2020 report from UNICEF about Georgian YWLS and a prior study with Georgian YWLS (see pages 5-6).

Reviewer 2: We were pleased that Reviewer 2 judged the paper as excellent and a pleasure to read. They made several minor comments and provided two suggestions to strengthen the publication, which we respond to below. 

1. Reviewer 2 asked that we review the manuscript to ensure there were spaces between words and references and provided an illustrative example from the original submission.

We have edited the revised manuscript to ensure appropriate spacing (see page 1).

2. The Reviewer pointed out that we were missing the word ‘life” in the following sentence "Currently, social service delivery for YWLS in Georgia focuses on both preventing youth from entering street life and on reintegrating young people into mainstream social [4]."

We have added the missing word “life” to this sentence.

3. Reviewer 2 asked us to edit the introduction to more concise, which would help to engage a wider audience.

We have made considerable changes to the introduction. Specifically, we edited the introduction on YWLS in Georgia and literature review on stigma. In so doing, we have cut approximately one page from the introduction (please see pages 1-6).

4. Reviewer 2 requested additional information on how the interview guide was developed. 

The interview guide was based on a literature review of studies and reports on YWLS in Georgia and with feedback from an advisory board that included community members involved with street connected youth. This information can be found on page 8. 

5. The Reviewer requested that be more explicit in the results on how provider stigma towards ethnic minority YWLS is tied to the ways in which youth and their families are seen as deviating from traditional conceptions of family drawn from patriarchal norms. 

We appreciate this suggestion and agree that patriarchy is likely an important factor that facilitates stigma towards ethnic minority YWLS and their families. Although we had briefly mentioned patriarchy in the prior version, we have elaborated on how provider’s statements reflect patriarchal values in in the revised results (see pages 14-17). 

6. The Reviewer also asked us to elaborate on the role of patriarchy in facilitating provider stigma towards ethnic minority YWLS in the discussion section. 

We have added a brief paragraph on the role of patriarchy in facilitating provider stigma towards ethnic minority YWLS in the discussion section. Here, we situate our results in the broader on literature elucidating patriarchal culture in Georgia (see page 

7. Finally, Reviewer 2 noted that they were left wondering if we had thoughts on the types of interventions or approaches could be taken to reduce stigma. 

We appreciate the Reviewer’s focus on interventions, as this work is part of a pilot study that we hope will lead to future interventions. At this time, we do not yet know what format or content should be targeted, as additional research is needed to identify the primary correlates of stigma amongst providers and the public that could serve as intervention targets. However, we believe that future interventions must be address intersectional stigma, target multiple social-ecological levels, and involve youth directly, ideally through youth participatory action research. We have mentioned this and the potential to learn from the global literature on mental illness stigma interventions on page 25 of the revised manuscript. 

We thank you and the Reviewers for the comments and opportunity to revise the manuscript. We believe the revised manuscript is stronger and hope that it is now ready for publication in PLOS One.

Sincerely,

Shorena Sadzaglishvili, PhD, MSW

Professor of Social Work

Ilia State University

Attachment Submitted filename: Response to Reviewers sh.docx Click here for additional data file.

# Peer review: Decision Letter 1

**DOI:** 10.1371/journal.pone.0286710.r003

**Authors:** Olujide Arije

20 Jan 2023

PONE-D-22-13919R1“They walk with bare feet on broken glass, they are not developing normally, and they are not learning anything”: Provider perspectives on stigma towards native and ethnic minority street-connected youth in the Republic of GeorgiaPLOS ONE

Dear Dr. Sadzaglishvili,

Thank you for submitting your manuscript to PLOS ONE. After careful consideration, we feel that it has merit but does not fully meet PLOS ONE’s publication criteria as it currently stands. Therefore, we invite you to submit a revised version of the manuscript that addresses the points raised during the review process.

Well-done on the good work in responding to the earlier reviewers’ queries and comments. There are a few issues not captured by the earlier reviewers that are now presented below for your consideration. Please note that exact quotations are lifted from your manuscript to help contextualize the comments given.

**Introduction**

*1.* *“Although some research has examined the social, health, and economic needs of YWLS in Georgia [4], few studies have explored how providers perceive, manage, and respond to the stigma directed towards their beneficiaries.”*

Also

*“In a qualitative study with providers serving sex workers, providers described encountering courtesy stigma from friends and family, other providers, and the broader public [32].”*

What kind of providers/providers of what? It is useful for the authors to explain who providers are before using the expression without any qualification i.e. include a sentence on who the authors are referring to as providers early on in the manuscript.

*2.* *“The present study helps to address this gap by conducting in-depth interviews with 22 social service providers who serve YWLS in Tbilisi and Rustavi, two major cities home to an estimated 1,094 YWLS [11].”*

The expectation here would be a statement of the study objective not of the data collection method.

*3.* *“These groups are based in major urban centers and led by State Senior Social Workers, with allied health professionals like psychologists, managers, peer educators, and logistics officers.”*

Managers of what?

1. It is not necessary to present subtitles in the introduction.

*5.* *“To date, little research has examined provider perspectives on the role of stigma in delivering services to diverse groups of YWLS in the Georgian context.”*

This is a repetition from a previous section.

*6.* *“The present study helps to address these gaps by analyzing qualitative data from in-depth interviews conducted with key informants (n=22) employed in social service organizations working with YWLS in Tbilisi and Rustavi, Georgia.”*

Including this detail of methodology in the introduction is not appropriate. It is more important to state the main objective of the research. Moreover, an earlier statement appeared to give this same information. So, in addition to being inappropriate in the introduction, there is a repetition of the same information in the introduction.

Consider replacing with this statement:

*“The aim of this study was to assess the perspectives of social service providers for YWLS on stigma towards native and ethnic minority street-connected youth in the Republic of Georgia.”*

**Method**

1. This section should start with a statement about the study design, followed by the study settings/location. This can then be followed by descriptions of the study populations/participants.

*2.* *“Key informants did not have an official relationship with the study and were not involved in developing the study or interpreting study results.”*

Consider replacing with:

“None of the study participants had official relationship with the study and were not involved in developing the study or interpreting its results.”

3. as the creation of the study guide occurs separately and before data collection

*4.* *“When appropriate, interviewers also used probes to elicit additional details from KIs”*

The question one would ask is when is it not appropriate to use probe in open-ended interview sessions. Probing is indeed one of the cardinal interview skills in qualitative research.

1. Please be specific about the language in which interviews were conducted and the process of translation if there was any.
2. Positionality:

*“This process was especially useful when talking about the presence of cultural* *racism* *directed towards ethnic minorities in Georgia, as this manifestation of stigma is both similar and distinct from racism within the United States”*

The term racism is being introduced for the first time in the manuscript but there is no indication in the introduction that this study was directed at issues about racism, at least it wasn’t expressly stated

**Result**

1. The second paragraph of the result presents the five core themes as found in this study. However, from a stylistic perspective this is presentation of a summary of the result before presenting the results in a section that should immediately present the result. This paragraph is not appropriately placed. In fact, some authors list their core themes under data analysis and expand on them on the results section. I suggest that this particular paragraph be reworked into the summary of the whole work.
2. The claim of cultural racism is not immediately justifiable as presented because there is no indication of the ethnicity of the provider in the immediate context, unless the authors can clearly state that there are no ethnic minorities among the providers, which doesn’t seem to be the case.
3. *“Finally, we found that a subset of providers who discussed public stigma towards YWLS reported negative attitudes and beliefs about ethnic minority”*

How many were in this subset of providers? For instance, can authors say how many of the 22 persons interviewed had this attitude, and describe commonalities among this group. While frequency is not a focus in qualitative research, it becomes significant when there are weighty claims. Authors should demonstrate that this is not a chance finding and that there are some commonalities among those who seemed to express racism.

It was in the discussion (see below) that the authors presented these facts. This is not transparent enough.

*“Notably, provider stigma towards ethnic minority YWLS was observed among a number of staff with advanced educational degrees, e.g., social workers and psychologists, as well as peer educators—young people with their own history of working and/or living on the street. Prior research has found that provider stigma can persist in the face of education [40,41]”*

1. *“Analysis of provider data revealed that YWLS are viewed as having a “spoiled identity,” from which labeling, stereotyping, separation and discrimination follows [14,15]. “*

This should be a discussion point, it is not a result from this study.

1. *“Providers also detailed how public stigma manifested within different public institutions to*
*exclude youth from accessing a range of services and entitlements. *

*Institution stigma was especially prevalent …”*

Close the paragraph between the above

1. *“Institutional stigma was especially prevalent in schools”*

Prevalence is a quantitative term. Use a more qualitative term.

Consider: “Stigma was reported to be common in schools”

Also, authors should be careful to report what the interviews said rather than their own interpretation of what was said. Institutional stigma is a technical term, and if it wasn’t used by the study participants then the authors should not use it in the result. However, in the discussions the authors can go on to identify the stigma observed as institutional.

1. Authors need to reconsider how they work quotations into narratives. In many places, it didn’t read well both grammatically and stylistically as presented. Consider the example below:

*“Psychologist explained why school administrators are reluctant to enroll YWLS because their mere presence is thought to spoil the school: “...in most cases, there is less tolerance, and they think it's something: ‘I don't want to ruin my school.’.”*

Suggested rewrite:

A psychologist participant explained that some school administrators were reluctant to enroll YWLS saying, for instance, *“…I don't want to ruin my school…”,* because the mere presence of YWLS in the school was thought would ‘spoil’ the school.

Note that spoil is in inverted commas

1. *“In addition to overt attempts to exclude YWLS from schools, providers noted that schools were*
*often hostile places for YWLS, with many being bullied by other youth, or experiencing hostility*
* from administrators, teachers and parents of other youth.”*

Rather than provide quotation for use of law twice, provide quotation for the point being made about schools being hostile for YWLS. This seems a point too important to gloss over.

1. *“Cultural racism produces intersectional stigma for ethnic minority YWLS”*

The authors assume every reader would understand or can define terms Cultural racism and intersectional stigma. Authors need to reserve the type of value judgement seen here for discussion where they can define these terms, or define their use of the terms earlier in the text e.g. in the introduction. Other terms used without explaining include institutional stigma and courtesy stigma

1. *“In their responses, providers endorsed patriarchal gender norms by framing ethnic minority YWLS and their families as deviating from traditional family structures.”*

This is a matter of the interpretation of the authors that should be in discussion not results. Furthermore, it seems to be a big leap from participants narrating their lived experiences and calling such experiences racist. For instance, the scenario presented in the quotation below describes what the speaker has seen.

This study is focused on children of ethnic minority but there is no indication that this is not the case with children who are not ethnic minorities. For instance; the quotation below from one of the study participants could be the experience of any child that lives on the street regardless of ethnicity

*“For Roma children, mental retardation is more common among them because they grow up on the street...Mental retardation is more common among Azerbaijan-Kurdish children, because they grow up actively on the street, they are born on the street. If you go to Africa, you will be able to see the baby who is naked outside, and there are pieces of glass on the ground and the baby walks barefoot. That is, they grow up in an environment where they cannot develop and learn. They have the same life. They go*

*outside in the morning and come home and fall asleep in the evening. They can't eat, they don't know how to wash their hands if we do not help.... there is one child also in the street, who is a Kurdish child and when you ask something, it takes a long time for him to answer your question. I don't know what to call it, what kind of diagnosis, but generally, it is a mental retardation because they grow up in an environment where they cannot learn.”*

Also consider the quotation below:

*“It is very common that Azeri-Kurdish and Roma girls of 13 start their sexual lives. It is mandatory to marry, have children and beg in the streets with children.... Their communities do not consider child marriages as criminal activity and a violation of a child’s rights.”*

Is this statement untrue in any respect? Authors should only have labeled this quotation a negative stereotype if they can further demonstrate from literature that such claims are, in fact, untrue. An argument can be made here that this was a description of an unfortunate and bad circumstance, but it does not translate directly that the person describing the scenario is implicitly racist.

1. *For many youth, self-stigma manifested as feelings of shame, negative self-worth, and a heightened sense being different from other youth, e.g., “They are ashamed because they think they are behind, as they never went to school. They are different than other children and it affects everything” (Manager, Respondent 13).*

Consider rewording as: “For many youth, self-stigma was said to manifest as feelings of shame, negative self-worth….”

This is because it was not the children that were interviewed

Generally, the authors need to write their narratives as in a way that they are expressing the opinions of the key informant not of the YWLS

1. Note Inappropriate capitalization e.g. Coordinator, Manager etc.
2. One question of interest is: Are there children on the street that are not ethnic minority and is there any inclination that their experience is different from those that ethnic minorities? Why have the authors not assessed issues around all YWLS but focused on ethnic minority?

**Discussion**

1. *“The processes described by providers follow those originally outlined by Goffman [15].”*

It is better to give the outline of the process than allude to it.

1. *“We also find support for the presence of intersectional [21], institutional, and structural stigma [43].”*

As already mentioned, authors have not defined any of these terms in the manuscript.

1. *“Importantly, providers did not report elevated stigma towards YWLS who are from Russia or*
*other European countries, who also are part of the population of YWLS in Georgia [4,65].”*

It will be interesting to know from literature if persons of Romani or Azeri-Kurdish origin who are not living on the street experience any form of stigmatization/discrimination by the virtue of their places of origin.

1. *“Although providers clearly recognized stigma as a negative animating force in the lives of YWLS, especially those from ethnic minority groups, a subset of providers also endorsed*
*stigmatizing beliefs towards ethnic minority youth.”*

Again, the argument of a subset of providers need to be made strong. Subset implies that there is some common underlying characteristics, So, one might expect age, profession, ethnicity etc. If there is no commonalty among those with the said opinion, then they are not really subset in the context of this research. In that case the authors would need to reword their statements to read more appropriately like: *“…some of the providers seemed to endorse stigmatizing beliefs…”*

1. *“We observed the same discourse among providers in their descriptions of Roma children being exposed to dangerous family environments.”*

Have the authors considered way that living on the street irrespective of ethnicity exposes to dangerous living experiences which may include a dangerous family environment. Some of these lack of clarity about causality as well as definition of terms would be cleared up with a conceptual framework that shows the dynamics of stigma for YWLS

1. *“Notably, provider stigma towards ethnic minority YWLS was observed among a number*
*of staff with advanced educational degrees, e.g., social workers and psychologists, as well as peer educators—young people with their own history of working and/or living on the street.”*

Again, this information did not come up in the result.

1. *“It is possible that the negative views displayed here shape who providers engage in services, highlighting another pathway through which stigma may shape the social exclusion of ethnic minority YWLS.” *

There is no indication at from the data presented that any provider acted in the way claimed here. Generalization should not be made beyond the scope of the data available. 

1. *“Our study adds to the growing body of literature on the role of courtesy stigma [32–34]”*

Again, key terms are not defined anywhere in the manuscript

As a general comment, a major limitation in this study is that there was no focus on YWLS of non-minority ethnicity. So, it cannot be inferred from this study if the experience of minority YWLS described is unique, or other categories of YWLS also experience similar stigma.

Please submit your revised manuscript by Mar 06 2023 11:59PM. If you will need more time than this to complete your revisions, please reply to this message or contact the journal office at plosone@plos.org. When you're ready to submit your revision, log on to https://www.editorialmanager.com/pone/ and select the 'Submissions Needing Revision' folder to locate your manuscript file.

Please include the following items when submitting your revised manuscript:

- A rebuttal letter that responds to each point raised by the academic editor and reviewer(s). You should upload this letter as a separate file labeled 'Response to Reviewers'.
- A marked-up copy of your manuscript that highlights changes made to the original version. You should upload this as a separate file labeled 'Revised Manuscript with Track Changes'.
- An unmarked version of your revised paper without tracked changes. You should upload this as a separate file labeled 'Manuscript'.

If you would like to make changes to your financial disclosure, please include your updated statement in your cover letter. Guidelines for resubmitting your figure files are available below the reviewer comments at the end of this letter.

If applicable, we recommend that you deposit your laboratory protocols in protocols.io to enhance the reproducibility of your results. Protocols.io assigns your protocol its own identifier (DOI) so that it can be cited independently in the future. For instructions see: https://journals.plos.org/plosone/s/submission-guidelines#loc-laboratory-protocols. Additionally, PLOS ONE offers an option for publishing peer-reviewed Lab Protocol articles, which describe protocols hosted on protocols.io. Read more information on sharing protocols at https://plos.org/protocols?utm_medium=editorial-email&utm_source=authorletters&utm_campaign=protocols.

We look forward to receiving your revised manuscript.

Kind regards,

Olujide Arije

Academic Editor

PLOS ONE

Journal Requirements:

Please review your reference list to ensure that it is complete and correct. If you have cited papers that have been retracted, please include the rationale for doing so in the manuscript text, or remove these references and replace them with relevant current references. Any changes to the reference list should be mentioned in the rebuttal letter that accompanies your revised manuscript. If you need to cite a retracted article, indicate the article’s retracted status in the References list and also include a citation and full reference for the retraction notice.

[Note: HTML markup is below. Please do not edit.]

Reviewers' comments:

Reviewer's Responses to Questions

**Comments to the Author**

1. If the authors have adequately addressed your comments raised in a previous round of review and you feel that this manuscript is now acceptable for publication, you may indicate that here to bypass the “Comments to the Author” section, enter your conflict of interest statement in the “Confidential to Editor” section, and submit your "Accept" recommendation.

Reviewer #2: All comments have been addressed

**********

2. Is the manuscript technically sound, and do the data support the conclusions?

The manuscript must describe a technically sound piece of scientific research with data that supports the conclusions. Experiments must have been conducted rigorously, with appropriate controls, replication, and sample sizes. The conclusions must be drawn appropriately based on the data presented. 

Reviewer #2: Yes

**********

3. Has the statistical analysis been performed appropriately and rigorously? 

Reviewer #2: N/A

**********

4. Have the authors made all data underlying the findings in their manuscript fully available?

The PLOS Data policy requires authors to make all data underlying the findings described in their manuscript fully available without restriction, with rare exception (please refer to the Data Availability Statement in the manuscript PDF file). The data should be provided as part of the manuscript or its supporting information, or deposited to a public repository. For example, in addition to summary statistics, the data points behind means, medians and variance measures should be available. If there are restrictions on publicly sharing data—e.g. participant privacy or use of data from a third party—those must be specified.

Reviewer #2: Yes

**********

5. Is the manuscript presented in an intelligible fashion and written in standard English?

PLOS ONE does not copyedit accepted manuscripts, so the language in submitted articles must be clear, correct, and unambiguous. Any typographical or grammatical errors should be corrected at revision, so please note any specific errors here.

Reviewer #2: Yes

**********

6. Review Comments to the Author

Please use the space provided to explain your answers to the questions above. You may also include additional comments for the author, including concerns about dual publication, research ethics, or publication ethics. (Please upload your review as an attachment if it exceeds 20,000 characters)

Reviewer #2: This is excellent work and the authors have addressed all my comments satisfactorily. I look forward to seeing this article published.

**********

7. PLOS authors have the option to publish the peer review history of their article (what does this mean?). If published, this will include your full peer review and any attached files.

If you choose “no”, your identity will remain anonymous but your review may still be made public.

**Do you want your identity to be public for this peer review?** For information about this choice, including consent withdrawal, please see our Privacy Policy.

Reviewer #2: No

**********

[NOTE: If reviewer comments were submitted as an attachment file, they will be attached to this email and accessible via the submission site. Please log into your account, locate the manuscript record, and check for the action link "View Attachments". If this link does not appear, there are no attachment files.]

While revising your submission, please upload your figure files to the Preflight Analysis and Conversion Engine (PACE) digital diagnostic tool, https://pacev2.apexcovantage.com/. PACE helps ensure that figures meet PLOS requirements. To use PACE, you must first register as a user. Registration is free. Then, login and navigate to the UPLOAD tab, where you will find detailed instructions on how to use the tool. If you encounter any issues or have any questions when using PACE, please email PLOS at figures@plos.org. Please note that Supporting Information files do not need this step.

# Author comment: Author response to Decision Letter 1

**DOI:** 10.1371/journal.pone.0286710.r004

20 Feb 2023

February 17, 2023

PONE-D-22-13919R1

“They walk with bare feet on broken glass”: Provider perspectives on stigma towards native and ethnic minority street-connected youth in the Republic of Georgia

Dear Dr. Arije,

Thank you for the opportunity to further revise our manuscript. We appreciate that the original Reviewers determined that we had responded to their primary concerns. We also welcome the opportunity to further strengthen the purpose, findings, and relevance of the study. Below, please find our responses to your comments and questions. In most cases, we made the requested changes. In some cases, however, the requested change or clarification was already present in the manuscript; in order to avoid repetition, we did not add duplicate information to the revised manuscript but rather sought to clarify the requested information. As instructed, we have made all edits in Track Changes. 

Introduction

1. The Editor asked to clarify the type of providers we interviewed and are referring to in the manuscript. They pointed out two specific passages and requested we clarify the type of providers early in the manuscript:

“Although some research has examined the social, health, and economic needs of YWLS in Georgia [4], few studies have explored how providers perceive, manage, and respond to the stigma directed towards their beneficiaries.

“In a qualitative study with providers serving sex workers, providers described encountering courtesy stigma from friends and family, other providers, and the broader public [32].”

In the revised manuscript, we have clarified that we are referring to social service providers who deliver services to YWLS in both the abstract and introduction. In addition, we have clarified the types of providers in the literature review. For example, for citation 32, this was health service providers that included psychologists, nurses, drug counselors and physicians (see page 5). 

2. The Editor noted that the following sentence states the method and not the study objective: “The present study helps to address this gap by conducting in-depth interviews with 22 social service providers who serve YWLS in Tbilisi and Rustavi, two major cities home to an estimated 1,094 YWLS [11].” 

In the revised manuscript, we have edited the sentence on page 2 to state: “The present study helps to address this gap by examining how social service providers who serve YWLS in Tbilisi and Rustavi, two major cities home to an estimated 1,094 YWLS [11], perceive the social contexts in which they deliver services to YWLS.”

3. The Editor requested that we clarify the types of managers described in the following sentence: “These groups are based in major urban centers and led by State Senior Social Workers, with allied health professionals like psychologists, managers, peer educators, and logistics officers.” On page 3 of the revised manuscript, we now state that we are referring to managers of social service organizations.

4. The Editor noted that it is not necessary to present subtitles in the introduction. As requested, we have deleted all subtitles from the introduction.

5. The Editor requested that we delete the following sentence, as it repeated a prior statement: “To date, little research has examined provider perspectives on the role of stigma in delivering services to diverse groups of YWLS in the Georgian context.” We have deleted this sentence. 

6. The Editor noted that we conflated the study objective with study methods, as evidenced in the following sentence: “The present study helps to address these gaps by analyzing qualitative data from in-depth interviews conducted with key informants (n=22) employed in social service organizations working with YWLS in Tbilisi and Rustavi, Georgia.” They requested we consider replacing with this statement: “The aim of this study was to assess the perspectives of social service providers for YWLS on stigma towards native and ethnic minority street-connected youth in the Republic of Georgia.” 

We appreciate the Reviewer’s point. However, the purpose of our study was not to explore stigma but to explore provider perspectives on the social contexts in which they serve YWLS. Themes related to stigma and the ways in which providers perceive differential stigma among native and ethnic minority youth emerged from the data. Nevertheless, we agree that we have conflated the study objectives with study methods and have amended the sentence to note that the objective of the study was to better understand how diverse social service providers perceive the social contexts in which they deliver services to street-connected youth (see page 7).

Method

7. The Editor noted: “This section should start with a statement about the study design, followed by the study settings/location. This can then be followed by descriptions of the study populations/participants.” 

On page 8, we have now added a description of the study design to the first sentence of the Method section.

8. The Editor requested that we consider replacing this sentence “Key informants did not have an official relationship with the study and were not involved in developing the study or interpreting study results” with: “None of the study participants had official relationship with the study and were not involved in developing the study or interpreting its results.” This change has been made in the revised manuscript (see page 8).

9. The Editor requested we clarify our use of probes, based on the following sentence: “When appropriate, interviewers also used probes to elicit additional details from KIs” They noted that one should ask when is it not appropriate to use probes in open-ended interview sessions, as probing is a cardinal interviewing skill in qualitative research.

We agree that probing is a fundamental interviewing skill in qualitative research. However, when respondents provide thick descriptions that fully answer a question, it may not be necessary to use a probe. Rather, probes were selectively used to elicit information when responses were vague or unclear. Nevertheless, we have deleted this sentence so as to avoid any confusion on the part of readers. 

10. The Editor asked us to please be specific about the language in which interviews were conducted and the process of translation if there was any. This information was included in the original submission: all interviews were conducted in Georgian (see page 9) and translation was conducted using the Euro-Reves method. We now briefly describe the translation method on page 10. 

11. The Editor requested clarity about our use of the term cultural racism in our Positionality section, where we stated: “This process was especially useful when talking about the presence of cultural racism directed towards ethnic minorities in Georgia, as this manifestation of stigma is both similar and distinct from racism within the United States.” The Editor noted that the term racism was being introduced for the first time in the manuscript but there was no indication in the introduction that this study was directed at issues about racism. 

The study was not directed at racism but rather at understanding how social service providers understand the social context of service delivery to YWLS. Through the analysis of the qualitative data, we identified the broad theme stigma. Within stigma, we then identified sub-themes, including the ways in which providers report their perceptions of stigma towards ethnic minority YWLS. We then drew on a large body of research that has documented cultural racism towards Roma groups. In the revised manuscript, we have attempted to address the Editor’s concerns in several ways. First, in the introduction to the manuscript, we have expanded our discussion on the specific forms of stigma and discrimination experienced by Romani groups (see page 6). Whereas the prior submission briefly mentioned cultural racism, we now define both antiziganism and Romaphobia, drawing on literature that conceptualizes these phenomena as forms of cultural racism that are connected to structural stigma. We believe these changes better prepare readers for our mention of cultural racism in the positionality section. 

Results

12. Although the Editor noted that this was a stylistic preference, they felt that the summary of the themes we presented in the results was not appropriately placed. They noted that some authors place such a summary in their data analysis section and asked that we rework this particular paragraph into the summary of the whole work.

We agree with the Editor that this is a stylistic point. Nevertheless, we have attempted to be responsive to the Editor’s comment by placing the summary of the results in the data analysis section. It now proceeds the positionality section. In addition, we have ensured that the summary follows the order of the results. Please see page 10.

13. The Editor stated: “The claim of cultural racism is not immediately justifiable as presented because there is no indication of the ethnicity of the provider in the immediate context, unless the authors can clearly state that there are no ethnic minorities among the providers, which doesn’t seem to be the case.”

The Editor rightly notes that the sample was not exclusively Georgian, with a small number of respondents identifying as Azeri-Kurdish or Romani. However, we do not include the ethnicity of providers because doing so would jeopardize the anonymity and confidentiality of the providers, who could be deductively identified by providing both ethnicity and professional role given the overall small sample and community of local organizations and providers working to support YWLS. We politely disagree with the Editor that it is not possible to infer some level of cultural racism from the data. First, some providers described their beliefs that ethnic minority youth are subjected to negative attitudes or discrimination based on their perceived ethnic membership or on the color their skin. In addition, there is a large volume of prior research documenting high levels of anti-ziganism directed towards Romani groups, much of which is situated within the context of cultural racism. Thus, the statements from providers about ethnic minority youth are not without context. In response to the Editor’s concerns, we have expanded our discussion of anti-ziganism and Romaphobia in the introduction (page 6). We also have included additional quotes from providers, where they compare and contrast different groups of youth. In the results and discussion, we have ensured that none of our statements are causal and that providers are reporting their perceptions. In the results, we now title the section Intersectional stigma and have deleted the phrase about cultural racism producing intersectional stigma (see page 14).

14. The Editor requested that we not use the term subset in the following sentence: “Finally, we found that a subset of providers who discussed public stigma towards YWLS reported negative attitudes and beliefs about ethnic minority.” The Editor then stated: “How many were in this subset of providers? For instance, can authors say how many of the 22 persons interviewed had this attitude, and describe commonalities among this group. While frequency is not a focus in qualitative research, it becomes significant when there are weighty claims. Authors should demonstrate that this is not a chance finding and that there are some commonalities among those who seemed to express racism.” 

The Editor rightly notes that there are different perspectives on whether qualitative results should be quantified. We agree and have attempted to demonstrate the strength of our findings by listing the relevant quotes under the identified themes, the professional role of the respondent, and respondent number. As requested, we have removed the use of the word subset. We also have provided additional data on the ways in which providers discussed ethnic minority youth. We do not believe that the finding is a chance one, as providers did not tend to describe Georgian or other YWLS with similar language; we have moved that statement from discussion to page 16 of the results. 

15. The Editor did not think we were being transparent enough with this statement: “Notably, provider stigma towards ethnic minority YWLS was observed among a number of staff with advanced educational degrees, e.g., social workers and psychologists, as well as peer educators—young people with their own history of working and/or living on the street. Prior research has found that provider stigma can persist in the face of education [40,41]”

This section was included in the discussion because we believed it was an interpretive point that was better suited for the discussion versus the results, a point raised by the Editor in another comment. In the revised manuscript, we now note that we included respondent role and study identification number as part of our coding (see page 10). 

16. The Editor requested that we modify the following sentence on page 12: “Analysis of provider data revealed that YWLS are viewed as having a “spoiled identity,” from which labeling, stereotyping, separation and discrimination follows [14,15].” They noted that it should be a discussion point, not a result from this study. This has been deleted from page 12 and is now included in the discussion on page 22.

17. The Editor noted an erroneous return between the sentence after: “Providers also detailed how public stigma manifested within different public institutions to exclude youth from accessing a range of services and entitlements.” This change has been made (see page 12).

18. The Editor requested we move away from quantitative language in the following sentence: “Institutional stigma was especially prevalent in schools,” and use a more qualitative term, such as “Stigma was reported to be common in schools.” This change has been made on page 12 of the revised manuscript and we have edited the manuscript to ensure this language is not present in other places. 

19. As a general comment, the Editor requested that we report what the interviewees said rather than our own interpretation of what was said. As an example, they noted that institutional stigma is a technical term and if it wasn’t used by the study participants, then we should not use it in the result. They recommended we include it in the discussion section instead. We have organized our themes to map on to larger theoretical constructs, like public, institutional, intersectional or courtesy stigma and have edited the results to ensure that it does not read like providers are using these terms. 

20. The Editor requested that we reconsider how we work quotations into narratives, noting that it didn’t read well both grammatically and stylistically. The Editor provided the following example: “Psychologist explained why school administrators are reluctant to enroll YWLS because their mere presence is thought to spoil the school: “...in most cases, there is less tolerance, and they think it's something: ‘I don't want to ruin my school.’” They requested the following edit: “A psychologist participant explained that some school administrators were reluctant to enroll YWLS saying, for instance, “…I don't want to ruin my school…,” because the mere presence of YWLS in the school was thought would ‘spoil’ the school. 

This reflected a typographical error (i.e., missing A at the start of the sentence). We have edited this sentence but did not make the complete change as the quote was expanded to address another request from the Editor to provide additional data on the ways in which schools were hostile to YWLS (see Item 21 below). In addition, we have reviewed and edited quotes throughout the manuscript. Often times, this included stating the role of the study participant in the introduction to the quote, rather than in the parentheses.

21. The Editor requested that we provide additional quotes for the statement that “In addition to overt attempts to exclude YWLS from schools, providers noted that schools were often hostile places for YWLS, with many being bullied by other youth, or experiencing hostility from administrators, teachers and parents of other youth.” They also asked us to consider deleting the quotes about how providers use the law to overcome school administrator’s refusal to enroll YWLS.

In the revised manuscript, we have edited this section of the results to provide additional quotes from providers on the ways in which they perceive schools to be hostile places for YWLS. The additional data appears in on pages 12-13, which is then followed by the two quotes about how providers rely on public laws and legal actors to enroll YWLS in schools. 

22. The Editor asked us to clarify the terms courtesy stigma, institutional stigma, cultural racism and intersectional stigma. In so doing, the Editor also asked us to reserve value judgements for discussion where we can define these terms, or to define our use of the terms earlier in the text, e.g., in the introduction. 

We included the definition of intersectional stigma on page 4 of the original manuscript in the following sentence: “Recent scholarship has focused on intersectional stigma, i.e., the presence of multiple devalued social markers, such as race, class, and sexual identity, that interact to produce layered experiences of stigma and discrimination [19–22].” Courtesy stigma was likewise defined on page 4 of the manuscript in the following sentence: “Because stigma is socially produced, public stigma can be transferred to people affiliated with stigmatized groups (i.e., courtesy stigma), including health and social service providers [32–34].” We also have elaborated on our original description of cultural racism in the manuscript. In the revised introduction, we now define antiziganism and Romaphobia, which has expanded the discussion of cultural racism (see page 6 of the revised manuscript). Finally, institutional and structural stigma are now defined on page 5. 

23. The Editor was concerned that we provided too much interpretation in the results section and asked us to delete the following sentence from the results: “In their responses, providers endorsed patriarchal gender norms by framing ethnic minority YWLS and their families as deviating from traditional family structures.” We have deleted this sentence. This interpretation also was deleted from the discussion section in light of other changes made to the revised manuscript. 

24. The Editor was concerned that we were making leaps from participants narrating their lived experiences to calling such experiences racist. The Editor asked us to label quotes in the results as negative stereotypes and to consider the extent to which there may be kernels of truth in provider’s statements that ethnic minority girls experience child marriages or begin their sex lives early. 

In the prior version, we interpreted these statements as forms of cultural racism because these stereotypes have been documented in numerous studies. We also are sensitive to the Editor’s point about labeling providers racist, which was not our intention. In the revised results, we use the language of negative stereotypes in the results and discussion. In the discussion, we have further contextualized providers’ stereotypes about ethnic minority youth in the broader literature. We also note that for some providers, there was a tendency to over-emphasize the role of families or cultures rather than the larger social contexts that are correlated with early marriage, early sex, and commercial sex—a phenomenon documented by numerous other scholars (see page 24).

25. The Editor asked us to edit the following sentence: “For many youth, self-stigma manifested as feelings of shame, negative self-worth, and a heightened sense being different from other youth, e.g., “They are ashamed because they think they are behind, as they never went to school. They are different than other children and it affects everything” (Manager, Respondent 13). to: “For many youth, self-stigma was said to manifest as feelings of shame, negative self-worth….” This change has been made (see page 20).

26. Related to the above point, the Editor requested that we edit the manuscript to clarify that we are discussing the opinions of the key informants and not of the YWLS. We have made this edit throughout the revised manuscript. 

27. The Editor asked us to use lowercase letters for job positions of respondents. We have made this change throughout the revised manuscript. 

28. The Editor posed several questions about the types of YWLS in Georgia, including: “Are there children on the street that are not ethnic minority and is there any inclination that their experience is different from those that are ethnic minorities? Why have the authors not assessed issues around all YWLS but focused on ethnic minority?”

In the original manuscript, we had described the different types of ethnic groups of youth who live and/or work on the street, including native Georgian, Russian, European, Azeri-Kurdish, and two distinct Romani groups of youth (pages 2-4). We discuss stigma towards ethnic minority YWLS in our findings because this emerged as a strong theme in the data. The interview questions did not ask providers about the differences between different groups of youth. Through our analysis, however, we identified that providers perceived that while all youth were subjected to public stigma, some reported that stigma and discrimination were worse for ethnic minority youth. The focus of this paper was thus driven by the data and the findings reflects the major themes that we identified in our thematic analysis. 

Discussion

29. Referring to the following sentence in the discussion, “The processes described by providers follow those originally outlined by Goffman [15].” The Editor stated that was better to give the outline of the process than allude to it. We outlined the processes in the introduction so did not repeat them here. In response, we have edited this sentence to include the labeling, stereotyping, separation and discrimination processes outlined by Goffman (see page 22).

30. In response to the following sentence, “We also find support for the presence of intersectional [21], institutional, and structural stigma [43].” the Editor stated: “As already mentioned, authors have not defined any of these terms in the manuscript.” Please see our response to item 22 above.

31. In response to this statement: “Importantly, providers did not report elevated stigma towards YWLS who are from Russia or other European countries, who also are part of the population of YWLS in Georgia [4,65],” the Editor noted that: “It will be interesting to know from literature if persons of Romani or Azeri-Kurdish origin who are not living on the street experience any form of stigmatization/discrimination by the virtue of their places of origin.” 

In the prior submission, we included a discussion of discrimination towards Romani in other countries. We have elaborated on this phenomenon to page 6 of the revised manuscript. In the revised manuscript, we now include this sentence at the end of the results section on page 16. In the discussion section, we now add a brief sentence on the need for future research on stigma towards Azeri-Kurds (see page 23). 

32. In response to the following statement, “Although providers clearly recognized stigma as a negative animating force in the lives of YWLS, especially those from ethnic minority groups, a subset of providers also endorsed stigmatizing beliefs towards ethnic minority youth,” the Editor stated: “Again, the argument of a subset of providers need to be made strong. Subset implies that there is some common underlying characteristics, So, one might expect age, profession, ethnicity etc. If there is no commonalty among those with the said opinion, then they are not really subset in the context of this research. In that case the authors would need to reword their statements to read more appropriately like: “…some of the providers seemed to endorse stigmatizing beliefs…”

We have removed the word subset and now state “some of the providers seemed to express negative beliefs…”

33. In response to the sentence that: “We observed the same discourse among providers in their descriptions of Roma children being exposed to dangerous family environments,” the Editor asked us to consider way that living on the street, irrespective of ethnicity, exposes youth to dangerous living experiences that may include a dangerous family environment. In addition, the Editor suggested we consider adding a conceptual framework that shows the dynamics of stigma for YWLS.

We agree with the Reviewer that living on the street or being in a dangerous family environment can expose youth to a host of negative living experiences, irrespective of their ethnicity; this is now noted on page 24. While we appreciate the Reviewer’s encouragement to develop a conceptual framework that shows the dynamics of stigma, we do not believe that we have sufficient data to develop such a framework. To develop a robust enough framework, we believe that we would need data from youth and from a larger sample of providers. Nevertheless, we are sensitive to the Editor’s comments. In the revised manuscript, we have expanded our discussion of the discursive framing of risk around ethnic minority YWLS (see page 24). In addition, we have edited the discussion to ensure that we are not drawing any causal inferences. Finally, we have expanded the recommendations on future research section to include longitudinal research than can help to elucidate the causal mechanisms between stigma and biopsychosocial health outcomes of YWLS over time (see page 27). 

34. The Editor stated that we did not provide evidence in the results to support the following statement: “Notably, provider stigma towards ethnic minority YWLS was observed among a number of staff with advanced educational degrees, e.g., social workers and psychologists, as well as peer educators—young people with their own history of working and/or living on the street.” 

In the results, we included the employment position for each quote, e.g., social worker, psychologist, outreach worker. In so doing, we are able to state that some of the social workers and psychologists, e.g., key informants with advanced degrees, and peer outreach workers, e.g., key informants with their own history of living and/or working on the street, did indeed express negative stereotypes, a type of stigma, towards YWLS. We disagree with the Editor that this information is not in the results. 

35. The Editor felt that some of the claims in the discussion extended beyond the scope of the data and highlighted this sentence as an example: “It is possible that the negative views displayed here shape who providers engage in services, highlighting another pathway through which stigma may shape the social exclusion of ethnic minority YWLS.” 

We were not attempting to claim that we observed this relationship in our data; rather, we were stating that this could be a potential pathway, which has been observed in prior work. In the revised manuscript, we have edited this sentence to be clear that we are not generalizing beyond the scope of the data (see page 25).

36. In response to the following statement, “Our study adds to the growing body of literature on the role of courtesy stigma [32–34],” the Editor wrote: “Again, key terms are not defined anywhere in the manuscript.” Please see our response to Item 22 above. 

37. The Editor stated: “As a general comment, a major limitation in this study is that there was no focus on YWLS of non-minority ethnicity. So, it cannot be inferred from this study if the experience of minority YWLS described is unique, or other categories of YWLS also experience similar stigma.”

We agree that a limitation of the study was that we did not include youth. We had previously mentioned this in the limitations section. To address the Editor’s concern, we have noted in the revised manuscript that future research with Georgian and ethnic minority YWLS is needed to better understand how stigma is experienced among each group (see page 26).

38. During the revision, we also reviewed for typographical and grammatical errors. We also would like to shorten the title of the manuscript to, “They walk with bare feet on broken glass,” as we believe the shorter title is more appropriate than the longer quote we originally had. 

We believe that the revisions have strengthened the manuscript and hope that the revised is now acceptable for publication. Thank you for your careful review.

Sincerely,

Shorena Sadzaglishvili, PhD, MSW

Professor 

Ilia State University 

ISU Room E327

Cholokashvili 3/5 Avenue

Tbilisi 0162, Georgia

Attachment Submitted filename: Response 2.20.23.docx Click here for additional data file.

# Peer review: Decision Letter 2

**DOI:** 10.1371/journal.pone.0286710.r005

**Authors:** Anastassia Zabrodskaja

15 Mar 2023

PONE-D-22-13919R2“They walk with bare feet on broken glass”: Provider perspectives on stigma towards native and ethnic minority street-connected youth in the Republic of GeorgiaPLOS ONE

Dear Dr. Sadzaglishvili,

Thank you for submitting your manuscript to PLOS ONE. After careful consideration, we feel that it has merit but does not fully meet PLOS ONE’s publication criteria as it currently stands. Therefore, we invite you to submit a revised version of the manuscript that addresses the points raised during the review process.

Please complete the revision following the instructions of the three reviewers.

Please submit your revised manuscript by Apr 29 2023 11:59PM. If you will need more time than this to complete your revisions, please reply to this message or contact the journal office at plosone@plos.org. When you're ready to submit your revision, log on to https://www.editorialmanager.com/pone/ and select the 'Submissions Needing Revision' folder to locate your manuscript file.

Please include the following items when submitting your revised manuscript:

- A rebuttal letter that responds to each point raised by the academic editor and reviewer(s). You should upload this letter as a separate file labeled 'Response to Reviewers'.
- A marked-up copy of your manuscript that highlights changes made to the original version. You should upload this as a separate file labeled 'Revised Manuscript with Track Changes'.
- An unmarked version of your revised paper without tracked changes. You should upload this as a separate file labeled 'Manuscript'.

If you would like to make changes to your financial disclosure, please include your updated statement in your cover letter. Guidelines for resubmitting your figure files are available below the reviewer comments at the end of this letter.

If applicable, we recommend that you deposit your laboratory protocols in protocols.io to enhance the reproducibility of your results. Protocols.io assigns your protocol its own identifier (DOI) so that it can be cited independently in the future. For instructions see: https://journals.plos.org/plosone/s/submission-guidelines#loc-laboratory-protocols. Additionally, PLOS ONE offers an option for publishing peer-reviewed Lab Protocol articles, which describe protocols hosted on protocols.io. Read more information on sharing protocols at https://plos.org/protocols?utm_medium=editorial-email&utm_source=authorletters&utm_campaign=protocols.

We look forward to receiving your revised manuscript.

Kind regards,

Anastassia Zabrodskaja, Ph.D.

Academic Editor

PLOS ONE

[Note: HTML markup is below. Please do not edit.]

Reviewers' comments:

Reviewer's Responses to Questions

**Comments to the Author**

1. If the authors have adequately addressed your comments raised in a previous round of review and you feel that this manuscript is now acceptable for publication, you may indicate that here to bypass the “Comments to the Author” section, enter your conflict of interest statement in the “Confidential to Editor” section, and submit your "Accept" recommendation.

Reviewer #2: All comments have been addressed

Reviewer #3: (No Response)

Reviewer #4: All comments have been addressed

**********

2. Is the manuscript technically sound, and do the data support the conclusions?

The manuscript must describe a technically sound piece of scientific research with data that supports the conclusions. Experiments must have been conducted rigorously, with appropriate controls, replication, and sample sizes. The conclusions must be drawn appropriately based on the data presented. 

Reviewer #2: Yes

Reviewer #3: Partly

Reviewer #4: Yes

**********

3. Has the statistical analysis been performed appropriately and rigorously? 

Reviewer #2: Yes

Reviewer #3: N/A

Reviewer #4: N/A

**********

4. Have the authors made all data underlying the findings in their manuscript fully available?

The PLOS Data policy requires authors to make all data underlying the findings described in their manuscript fully available without restriction, with rare exception (please refer to the Data Availability Statement in the manuscript PDF file). The data should be provided as part of the manuscript or its supporting information, or deposited to a public repository. For example, in addition to summary statistics, the data points behind means, medians and variance measures should be available. If there are restrictions on publicly sharing data—e.g. participant privacy or use of data from a third party—those must be specified.

Reviewer #2: Yes

Reviewer #3: No

Reviewer #4: No

**********

5. Is the manuscript presented in an intelligible fashion and written in standard English?

PLOS ONE does not copyedit accepted manuscripts, so the language in submitted articles must be clear, correct, and unambiguous. Any typographical or grammatical errors should be corrected at revision, so please note any specific errors here.

Reviewer #2: Yes

Reviewer #3: Yes

Reviewer #4: Yes

**********

6. Review Comments to the Author

Please use the space provided to explain your answers to the questions above. You may also include additional comments for the author, including concerns about dual publication, research ethics, or publication ethics. (Please upload your review as an attachment if it exceeds 20,000 characters)

Reviewer #2: (No Response)

Reviewer #3: Thank you for inviting me to review. This paper was previously reviewed, and per the letter from the manuscript authors, the reviewers indicated satisfaction with the response / edits. There were further comments / requests from the editors that the authors are now responding to in this version of the manuscript. There were many editor comments, and having reviewed the detailed responses from the manuscript authors, I think that they have adequately responded to them. (I agree that it was important more clearly connect the results / data with the interpretations / conclusions drawn by the authors. And the use of many terms related to stigma - e.g, courtesy stigma, intersectional stigma, cultural racism - needed more explanation / clear distinction, including how they were relevant for the results and implications.) The authors have made the great majority of the requested edits and adequately explained their thinking for the few edits they chose not to make.

Overall the topic of the paper is an interesting one - the role of governmental / social service institutions in addressing or augmenting stigma experienced by marginalized populations - and one not covered often / sufficiently in the global health literature. I think the points from the study highlighting how stigma is experienced by different groups, and that providers / social service institutions can reinforce some of the stigma - are worthwhile to document.

A few additional reflections for the authors to take under consideration are:

(1) I am not clear how the quote in the title sheds light on the main themes / points of the paper. I would pick another quote.

(2) This was already part of the author / editor exchange, but I am still not clear about the stated goal / objectives of this study with the phrase "provider perspectives on the social contexts surrounding the delivery of services." The term 'social contexts' is too vague to my mind, and would benefit from an example or a more specific phrase.

(3) The abstract uses the term 'courtesy stigma' which won't be clear to many readers unless you add an example or definition.

Reviewer #4: Once again, I appreciate the opportunity to review this detailed piece of writing. The area of study is one I am very passionate about. However, I noticed that a two-level extensive review has already been done on this manuscript, and most of the comments, corrections and observations by the reviewers are in order. I also noted that the authors had taken the time to address most of the concerns raised by the previous reviewers.

That said, my concern lies with the methodology section. The opening sentence doesn’t fit well with the first paragraph. The opening paragraph deals with the sampling plan, which ought not to be. The normal is to start with the research design, followed by the population of the study, the sampling plan, the method of data collection, the instruments used and then how the data were analysed.

From what I can gather from the paper, this is a qualitative, exploratory study. But the population from which the sample of 22 service providers was drawn is missing. It will be good to state the population. And what technique was adopted in sample selection? Can we say that the 22 participants were selected from a known population (total number of social service organisations)? If so, were they selected through randomisation, purposive or simply by availability? If the population is not known, how were organisations (agencies) selected and the participants identified – snowballing or through a documented list of service providers? I expect the writers to be specific here.

The choice of in-depth interviews with an interview guide comprising structured and unstructured questions is apt. The same goes for the choice of thematic analysis for data analysis. What is missing is the way these are presented in the paper. I, therefore, suggest that this section is reworked, adopting the recommended systematic approach. I believe that such an approach will make comprehension easier.

The other concern I have is the claim of “high” levels of stigma in the section on “Difficult working environments: provider encounters with courtesy Stigma”. Though this statement has been amended (deleted), it is truly difficult to showcase the level of significance in a qualitative study. However, there is a way out. You may use word cloud, which will clearly indicate the most prominent theme of keyword in your thematic analysis. This is in addition to using enough quotes from the participants. If you still wish to retain that statement (which, in my opinion, contributes significantly to the result, I suggest you present it in a word cloud.

**********

7. PLOS authors have the option to publish the peer review history of their article (what does this mean?). If published, this will include your full peer review and any attached files.

If you choose “no”, your identity will remain anonymous but your review may still be made public.

**Do you want your identity to be public for this peer review?** For information about this choice, including consent withdrawal, please see our Privacy Policy.

Reviewer #2: No

Reviewer #3: No

Reviewer #4: No

**********

[NOTE: If reviewer comments were submitted as an attachment file, they will be attached to this email and accessible via the submission site. Please log into your account, locate the manuscript record, and check for the action link "View Attachments". If this link does not appear, there are no attachment files.]

While revising your submission, please upload your figure files to the Preflight Analysis and Conversion Engine (PACE) digital diagnostic tool, https://pacev2.apexcovantage.com/. PACE helps ensure that figures meet PLOS requirements. To use PACE, you must first register as a user. Registration is free. Then, login and navigate to the UPLOAD tab, where you will find detailed instructions on how to use the tool. If you encounter any issues or have any questions when using PACE, please email PLOS at figures@plos.org. Please note that Supporting Information files do not need this step.

# Author comment: Author response to Decision Letter 2

**DOI:** 10.1371/journal.pone.0286710.r006

3 May 2023

April 21, 2023

PONE-D-22-13919R2

Anastassia Zabrodskaja, Ph.D.

Academic Editor

PLOS ONE

Dear Dr. Zabrodskaja,

Enclosed, please find the revision to Manuscript PONE-D-22-13919R2. We appreciate the Reviewers’ additional comments, and have attempted to respond to all suggestions. In most cases, we made the suggested changes. In one instance, a requested revision contradicted changes requested by the prior Academic Editor, who had provided a lengthy list of requested revisions. Nevertheless, we have made the requested change here, as we believe that the Reviewer’s suggestions improved the clarify of the manuscript. In total, there was only one suggestion that we were not able to pursue, and we note this below. Finally, as required, we have made all edits in Track Changes. The data remain available in the repository and we hope the changes address the different conclusions of the Reviewers about the conclusions and findings. 

Responses to Reviewers

Reviewer #3: We appreciate that Reviewer #3 determined that the paper addresses an understudied topic in the extant global health literature. We thank the Reviewer for the additional comments and opportunity to strengthen the manuscript. 

1. Reviewer 3 did not believe that the current quote, e.g., “they walk with bare feet on broken glass” shed light on the main themes of the paper and suggested we select another quote to use in the title. 

Thank you for this suggestion. We reviewed the manuscript and have selected a new quote for the title: “How can you kiss and touch this child and show affection towards her? What kind of woman are you?” We selected this quote because we believe that it reflects public stigma directed towards youth and courtesy stigma that providers report experiencing in their work with young people who live and/or work on the street.

2. The author requested that we better specify the term “social contexts” in the following phrase: “provider perspectives on the social contexts surrounding the delivery of services.” 

Thank you for this comment. On page 8 of the revised manuscript, we have added text to clarify this term. The sentence now reads: “The purpose of the interviews was to understand provider perspectives on the social networks and HIV prevention needs of YWLS, their experiences delivering services to YWLS, and their recommendations for developing trusting relationships with YWLS.” We also have clarified this aspect of the interview guide on page 10. 

3. The abstract uses the term 'courtesy stigma' which won't be clear to many readers unless you add an example or definition. We have added a definition of courtesy stigma to the abstract. 

Reviewer #4: We appreciate that Reviewer #4 deemed that we were responsive to the prior Editor’s comments and that the revisions had strengthened the rigor of the results and conclusions. We also welcome the Reviewer’s additional suggestions to strengthen the methodology section. 

1. Reviewer 4’s primary concern was with the methodology section. They stated that the “opening sentence doesn’t fit well with the first paragraph. The opening paragraph deals with the sampling plan, which ought not to be. The normal is to start with the research design, followed by the population of the study, the sampling plan, the method of data collection, the instruments used and then how the data were analysed.”

To address this concern, we have moved the paragraph titled “Present study” in the previous revision to the start of the methods. We also have edited this paragraph to address the Reviewer’s concern. As the Reviewer notes, this was a qualitative exploratory study. We now state this and have added the following paragraph to the start of the methods on pages 8-9.

“This qualitative exploratory study was conducted during the formative phase of See the Future in Us, a multi-method study examining the social networks and HIV prevention needs of YWLS [10]. During this exploratory phase, we conducted individual in-depth interviews with key informants employed in social service organizations working with YWLS in Tbilisi and Rustavi, Georgia. The purpose of the interviews was to understand provider perspectives on the HIV prevention needs of YWLS, their experiences delivering services to YWLS, and their recommendations for developing trusting relationships with YWLS. Key informants included social workers, psychologists, logistics officers who supported mobile outreach teams, managers of social service organizations, and peer educators who were young adults with a prior history of living and/or working on the street.”

2. The Reviewer asked us to better specify the information on the population from which the sample of 22 service providers was drawn, including the technique adopted in sample selection, whether the 22 participants were selected from a known population (such as the total number of social service organisations), and the selection method (e.g., randomisation, purposive, or convenience). If the population is not known, the Reviewer asked us to specify how the organisations were selected and how the participants identified, e.g., snowballing or through a documented list of service providers.

Thank you for the opportunity to clarify the sampling approach. We developed a sampling frame based on the 10 known governmental and non-governmental organizations that provide services to YWLS in Tbilisi and Rustavi. From this list, we used a purposive sampling method to identify and recruit potentially eligible key informants (KIs) who worked with YWLS in each agency. On page 9, we have revised the text to state:

“We developed a sampling frame based on the 10 known governmental and non-governmental organizations that provide services to YWLS in Tbilisi and Rustavi. From this list, we used a purposive sampling method to identify and recruit potentially eligible key informants (KIs) who worked with YWLS in each agency. KIs were eligible to participate if they (1) were 18 years or older, (2) worked in an agency providing services to YWLS; and (3) had direct contact with YWLS through their work. All eligible staff in each agency were contacted via telephone and invited to participate in an individual interview. During recruitment, the local research team explained the purpose of the study and all participants provided written informed consent. In total, 95% of all identified and eligible staff were recruited into the study. None of the study participants had an official relationship with the study and were not involved in developing the study or interpreting its results.”

3. Reviewer 4 stated: “The choice of in-depth interviews with an interview guide comprising structured and unstructured questions is apt. The same goes for the choice of thematic analysis for data analysis. What is missing is the way these are presented in the paper. I, therefore, suggest that this section is reworked, adopting the recommended systematic approach. I believe that such an approach will make comprehension easier.”

The presentation of the results have undergone considerable revisions during the review process. In our original submission, we had listed the themes at the start of the results. In the last revision, the prior Academic Editor requested we remove this text and just present the results. We are not aware of a recommended systematic approach to presenting the results and could not find one on the PLOS One webpages. However, we agree that the presentation of the results could be streamlined to improve reader comprehension. For this revision, the first and last authors reviewed prior articles published by PLOS One that included thematic analysis of qualitative data. Through reading these articles, we did not observe a single approach to presenting results. However, we noticed that most articles state the number of themes and subthemes at the start of the results, and list the primary themes—thereby providing readers with an early description of the results. In our revision, we have adopted this approach. To further strengthen the results, we revisited the results of the qualitative data analysis to enrich the titles for each theme, further grounding each theme in participant’s words. As we made these revisions, we also noticed that our subthemes were not clearly labeled; rather, they were included as part of a major theme. To further improve comprehension and clarity, we now clearly identify the subthemes that were present in relevant identified themes. On page 12, the revised introduction to the results now states: 

“We identified five themes and five subthemes. The primary themes are: (1) Already labeled objects who spoil everything: Public and institutional stigma directed towards YWLS; (2) Dirty and despicable criminals: Intersectional stigma for ethnic minority YWLS; (3) Provider stereotypes about ethnic minority youth: Labeling, stereotyping and separating “us” from “them;” (4) Youth fear, anxiety, and distrust: Provider perspectives on how public stigma affects YWLS; and (5) Difficult working environments: Provider encounters with courtesy stigma. Each theme and any relevant subthemes are discussed below, with illustrative quotes from interviews along with the professional role of each respondent.”

4. The final concern raised by Reviewer #4 regarded our previous claim of “high” levels of stigma in the section on “Difficult working environments: provider encounters with courtesy Stigma”. The Reviewer stated, “Though this statement has been amended (deleted), it is truly difficult to showcase the level of significance in a qualitative study. However, there is a way out. You may use word cloud, which will clearly indicate the most prominent theme of keyword in your thematic analysis. This is in addition to using enough quotes from the participants. If you still wish to retain that statement (which, in my opinion, contributes significantly to the result, I suggest you present it in a word cloud.”

We agree with the Reviewer that it is difficult to showcase the level of significance in a qualitative study. While we appreciate the suggestion to utilize a word cloud, we are not sure how to do this given that we coded the data in Georgian and would need to translate any word cloud. In reviewing the manuscript, we are comfortable with retaining the prior revision to not make this claim about high levels of stigma. We also have changed high to strong in other parts of the manuscript. 

We believe that the revisions have once again strengthened the manuscript and hope that the third revision is now acceptable for publication. Thank you, again, for the thoughtful reviews.

Sincerely,

Shorena Sadzaglishvili, PhD, MSW

Professor 

Ilia State University 

ISU Room E327

Cholokashvili 3/5 Avenue

Tbilisi 0162, Georgia

Attachment Submitted filename: Response to Reviewers FINAL PLOS One.docx Click here for additional data file.

# Editor report: Decision Letter 3

**DOI:** 10.1371/journal.pone.0286710.r007

**Authors:** Anastassia Zabrodskaja

23 May 2023

“How can you kiss and touch this child and show affection towards her? What kind of woman are you?”: Provider perspectives on stigma towards native and ethnic minority street-connected youth in the Republic of Georgia

PONE-D-22-13919R3

Dear Dr. Sadzaglishvili,

We’re pleased to inform you that your manuscript has been judged scientifically suitable for publication and will be formally accepted for publication once it meets all outstanding technical requirements.

Within one week, you’ll receive an e-mail detailing the required amendments. When these have been addressed, you’ll receive a formal acceptance letter and your manuscript will be scheduled for publication.

An invoice for payment will follow shortly after the formal acceptance. To ensure an efficient process, please log into Editorial Manager at http://www.editorialmanager.com/pone/, click the 'Update My Information' link at the top of the page, and double check that your user information is up-to-date. If you have any billing related questions, please contact our Author Billing department directly at authorbilling@plos.org.

If your institution or institutions have a press office, please notify them about your upcoming paper to help maximize its impact. If they’ll be preparing press materials, please inform our press team as soon as possible -- no later than 48 hours after receiving the formal acceptance. Your manuscript will remain under strict press embargo until 2 pm Eastern Time on the date of publication. For more information, please contact onepress@plos.org.

Kind regards,

Anastassia Zabrodskaja, Ph.D.

Academic Editor

PLOS ONE

Additional Editor Comments (optional):

Reviewers' comments:

# Editor report: Acceptance letter

**DOI:** 10.1371/journal.pone.0286710.r008

**Authors:** Anastassia Zabrodskaja

24 May 2023

PONE-D-22-13919R3 

“How can you kiss and touch this child and show affection towards her? What kind of woman are you?”: Provider perspectives on stigma towards native and ethnic minority street-connected youth in the Republic of Georgia 

Dear Dr. Sadzaglishvili:

I'm pleased to inform you that your manuscript has been deemed suitable for publication in PLOS ONE. Congratulations! Your manuscript is now with our production department. 

If your institution or institutions have a press office, please let them know about your upcoming paper now to help maximize its impact. If they'll be preparing press materials, please inform our press team within the next 48 hours. Your manuscript will remain under strict press embargo until 2 pm Eastern Time on the date of publication. For more information please contact onepress@plos.org.

If we can help with anything else, please email us at plosone@plos.org. 

Thank you for submitting your work to PLOS ONE and supporting open access. 

Kind regards, 

PLOS ONE Editorial Office Staff

on behalf of

Professor Anastassia Zabrodskaja 

Academic Editor

PLOS ONE
